# Student Medical Summit - Online 2022: Virtual. 5 February 2022

**DOI:** 10.1186/s12919-022-00235-w

**Published:** 2022-09-01

## A1. Targeting mutant p53 for the treatment of triple negative breast cancer: a pre-clinical study

### Anna Lawless^1^, Shane O’Grady^2^, Minhong Tang^2^, Michael J. Duffy^2,3^

#### ^1^UCD School of Medicine, University College Dublin, Belfield, Dublin, Ireland; ^2^UCD School of Medicine, Conway Institute of Biomedical and Biomolecular Research, University College Dublin, Belfield, Dublin, Ireland; ^3^UCD Clinical Research Centre, St. Vincent’s University Hospital, Dublin, Ireland

##### **Correspondence:** Anna Lawless (anna.lawless@ucdconnect.ie)

Triple negative breast cancer (TNBC) refers to an invasive subset of breast cancer that lacks oestrogen receptors (ER), progesterone receptors (PR) and lacks amplification of HER2 [1]. Thus, these patients cannot be treated with a targeted therapy and have poorer outcomes compared to patients with other subforms of breast cancer.

p53 it is the most frequently mutated gene in human cancer. Approximately 80% of patients with TNBC carry a p53 mutation. Recently, arsenic trioxide (ATO) was found to reactivate mutant p53 and convert it back to its normal wild-type form [2]. The aim of this research was to test if ATO might be a new treatment for TNBC. The ability of ATO to inhibit cell proliferation was determined using MTT assays while induction of apoptosis was measured using flow cytometry.

IC_50_ values for growth inhibition across 10 breast cancer cell lines ranged from 0.297-2.99 μM. Inhibition of proliferation was found to be independent of the cell line molecular subtype. No significant differences were found between IC_50_ values for TN vs non-TN cell lines (*p=0.597*) or between contact vs structural p53 mutants (*p=0.481*). For all cell lines investigated, ATO induced significant levels of apoptosis at a concentration of 5 μM.

Although our data are preliminary, we conclude that ATO is a potential new therapy for the treatment of p53 mutated cancer, including triple negative breast cancer. Since ATO is already approved for the treatment of acute promyelocytic leukaemia (APL), it should be straightforward to repurpose it for TNBC.

I would like to express my gratitude to my supervisor Professor Joe Duffy and all in the breast cancer research group in St. Vincent’s University Hospital. Thank you for all your encouragement and for giving me the opportunity to work with and learn from you.

**References**

1. Duffy MJ, Synnott NC, Crown J. Mutant p53 in breast cancer: potential as a therapeutic target and biomarker. Breast cancer research and treatment. 2018;170(2):213-9.

2. Chen S, Wu J-L, Liang Y, Tang Y-G, Song H-X, Wu L-L, et al. Arsenic Trioxide Rescues Structural p53 Mutations through a Cryptic Allosteric Site. Cancer cell. 2021;39(2):225-39.e8.

## A2. Transmissibility of COVID-19: in persons aged <5 years vs aged 5-12 years vs 12-18 years vs adults

### Elena Khoo^1^, Finbarr Condon-English^1^, Eoghan Mooney^1^, Michael Hanrahan^2^, Anne Sheahan^3^, Margaret O'Sullivan^3^, Mary O'Mahony^3^, Peter Barrett^2^, Philippa White^3^, Aline Brennan^2^, Orla Bruton^3^

#### ^1^School of Medicine, University College Cork, Ireland; ^2^School of Public Health, University College Cork. Ireland; ^3^Department of Public Health, HSE- South, Cork. Ireland

**Background**

SARS-CoV-2 has affected children and adolescents worldwide, with the pandemic taking a toll on their health and well-being. COVID-19 measures have disrupted the education of 1.6 billion students worldwide [1]. Children and adolescents are reported to have lower susceptibility to COVID-19 than adults, possibly attributed to their limited social interaction with mainly household members [2]. However, the full impact of community transmission among asymptomatic children requires further investigation.

**Methods**

A retrospective cohort study was used to examine the transmissibility of COVID-19 from primary cases to close contacts using data from Cork and Kerry, notified between 1/3/21 and 15/6/21. Cases were aged 0-4 (n=100), 5-11 (n=100), 12-17 years old (n=100) and these sub-groups were compared against unvaccinated adults.

This study uses extracted data from the National Case Tracker Customer Relationship Management (CRM) System. The effective R number and relative risk numbers were calculated and compared between the different subgroups.

**Results**

Cases aged 0-4 had 169 close contacts and infected 54% (95% CI 0.37 – 0.78; p <0.001) of people compared to unvaccinated adults. They had a 18% (95% CI 0.54- 1.24; p= 0.347) less risk of transmission to close contacts.

Cases aged 5-11 had 196 close contacts and had a 62% ( 95% CI 0.25 – 0.59; p < 0.001) lower rate of transmission. They had 50% (95% CI 0.31- 0.82; p= 0.005) less risk of transmission to close contacts.

Overall, there was no difference between the 12-17 and unvaccinated adults.

**Conclusion**

Our study found that 0-11 years old resulted in fewer secondary cases compared to unvaccinated adults. Transmission for cases aged 12- 17 were similar to adults except they led to fewer secondary cases outside of their household compared to unvaccinated adults.

**Acknowledgements**

Finbarr Condon- English, University College Cork

Eoghan Mooney, University College Cork

Michael Hanrahan, Department of Public Health, HSE-South, Cork, Ireland

**References**

1. New global tracker to measure pandemic’s impact on education worldwide [Internet]. Unicef.org. 2021 [cited 31 December 2021]. Available from: https://www.unicef.org/press-releases/new-global-tracker-measure-pandemics-impact-education-worldwide

2. Viner R, Mytton O, Bonell C, Melendez-Torres G, Ward J, Hudson L et al. Susceptibility to SARS-CoV-2 Infection Among Children and Adolescents Compared With Adults. JAMA Pediatrics [Internet]. 2021 [cited 31 December 2021];175(2):143. Available from: https://jamanetwork.com/journals/jamapediatrics/fullarticle/2771181

## A3. Senior sign-off in an Irish emergency department: is it feasible?

### Katie Ryan^1^, Emma-Louise Rogers^2^, John Cronin^2^, Conor Prendergast^2^

#### ^1^School of Medicine, University College Dublin, Dublin, Ireland; ^2^Department of Emergency Medicine, St. Vincent’s University Hospital, Dublin 4, Ireland

**Background**

Emergency Medicine (EM) clinicians are required to make critical decisions, often with limited information, resources, and time. Research has demonstrated that consultant-delivered care reduces waiting times, length of stay, number of admissions, and improves clinical outcomes [1]. Senior decision-making has been seen as a surrogate marker of the safety in the Emergency Department (ED). This audit was based on the Royal College of Emergency Medicine (RCEM) “Consultant Sign-Off” national audit which was carried out between the years 2011-2017 in the UK. This demonstrated an average rate of “Senior Sign-Off” of just 43% across four specified patient cohorts (three adult, one paediatric). This is the first audit to examine ‘Senior Sign-Off’ levels in an Irish ED.

**Method**

Data was collected retrospectively from the Maxims ED information system between July-October 2017, January-July 2020, and January-July 2021. Cycle 1 of this audit is pre-COVID-19, with cycle 2 and 3 occurring during the pandemic. Significant changes in staffing and methods for documentation occurred during this time.

Patient cohorts were selected using the RCEM ‘Consultant Sign-Off’ clinical standards [2]. As an adult-only ED, the paediatric cohort was not included in this audit:

1. Patients making an unscheduled return to the ED with the same complaint within 72h of discharge
2. Abdominal pain in patients >70 years
3. Atraumatic chest pain in patients >30 years

To reflect the ST4+ levels of clinician that were used in the UK, senior sign-off in this audit was defined as review by, or a case discussion with, an EM consultant, SpR, or senior registrar with 4+ years of EM experience. Only patients who were discharged from the ED were included.

After cycle 1, a teaching session was delivered to all ED clinicians. There was a move toward electronic note-keeping and the introduction of a designated doctor to document decisions made at clinical handovers with senior staff present. After cycle 2 a further teaching session was delivered and posters were placed around the ED to highlight the importance of documenting senior reviews and discussions.

**Results**

2,687 patients were included across the 3 cycles. The overall rate of documented Senior Sign-off across the three patient cohorts was 35% in cycle 1 (n=454), 62% in cycle 2 (n=1139), and 64% in cycle 3 (n=1194) (Table 1).

**Conclusion**

Significant improvements were made in the rate of documented ‘Senior Sign-Off’ of these high-risk patient cohorts across the three audit cycles. To ensure all ED patients, particularly those deemed “high-risk”, are seen by a senior doctor, an increase in ED consultant staffing is required.

**References**

1. Geelhoed GC, Geelhoed EA. Positive impact of increased number of emergency consultants. Arch Dis Child 2008; 93:62-64.

2. Boyle, A. Consultant Sign-Off. London, UK: The Royal College of Emergency Medicine; June 2016.

**Table 1 (abstract A3).** Documented senior sign-off rates across the 3 audit cycles

Cycle		N	Consultant (N, %)	ST4+ (N, %)	Senior sign-off (N, %)
**1**	**Total**	**454**	**36 (8%)**	**121 (27%)**	**157 (35%)**
	Unscheduled returns	114	9	26	
	Abdominal pain	63	3	13	
	Chest pain	277	24	82	
**2**	**Total**	**1139**	**232 (40%)**	**479 (42%)**	**711 (62%)**
	Unscheduled returns	138	31	54	
	Abdominal pain	112	32	48	
	Chest pain	889	169	377	
**3**	**Total**	**1094**	**272 (25%)**	**430 (39%)**	**701 (64%)**
	Unscheduled returns	181	36	81	
	Abdominal pain	76	16	33	
	Chest pain	837	220	316	

## A4. Microfluidic-microwave platforms for real-time, non-invasive and sensitive monitoring of bacteria and antibiotic susceptibility testing

### Rakesh Narang^1,2,3^, Sevda Mohammadhi^4^, Mehdi Mohammadi Ashani^1,2^, Mohammad Zarifi^4^, Amir Sanati-Nezhad^1,2,3^

#### ^1^BioMEMS and Bioinspired Microfluidic Laboratory, Department of Mechanical and Manufacturing Engineering, University of Calgary, Calgary, Alberta, Canada, ^2^Center for BioEngineering Research and Education, University of Calgary, Calgary, Alberta, Canada, ^3^Biomedical Engineering Graduate Program, University of Calgary, Calgary, Alberta, Canada, ^4^Okanagan Microelectronics and Gigahertz Applications (OMEGA) Lab, Faculty of Applied Science, Kelowna, BC, Canada

##### **Correspondence:** Rakesh Narang (rakesh.narang@ucdconnect.ie)

In 2019, the CDC reported over 2.8 million antibiotic-resistant bacterial infections in the United States [1]. Current clinical practices often require up to two weeks to accurately diagnose these infections and determine appropriate antibiotic courses for patients [2]. With contemporary methods of infection diagnosis and antibiotic susceptibility testing (AST) being time-consuming, expensive to operate, and lacking point-of-care (POC) testing potential in remote areas physicians are forced to over-prescribe broad-spectrum antibiotics, as diagnosis and AST are neglected [3]. Along with patients’ non-compliance in antibiotic therapy, pathogens subsequently develop resistances to multiple drugs [4]. Therefore, developing methods that improve the POC and high-throughput potential of infection diagnosis and AST practices is of critical need to treat patients more accurately, efficiently, and cost-effectively in hospitals and remote areas.

Microfluidics, lab-on-a-chip, and microelectromechanical systems (MEMS) are areas of studies highly valued for their user-friendly design, POC potential and high-throughput results, particularly in healthcare and biomedical sensing applications [5,6]. The current use of capillary forces to autonomously deliver fluids within microfluidic chips (Capillary Fluidics) has allowed microfluidic sensors to be integrated in POC and high-throughput sensors. Furthermore, the cheap and seamless integration of Capillary Fluidics with multiple sensory methods, such as optical, electrochemical and microwave sensing, makes it an attractive option to remedy the critical need of innovation in infection diagnosis and AST practices [7].

Here, microwave sensing was selected for its high sensitivity and inexpensive implementation with Capillary Fluidics to monitor pathogens and perform AST [8]. The microwave resonator generated electrical fields to detect dielectric shifts within the capillary microfluidic channels, allowing for the characterization of growing *E. coli* assays in a real-time, sensitive, and non-invasive manner for over 8 hours. Furthermore, two resonators were coupled in an array to enhance the sensor’s sensitivity and selectivity towards AST applications. The hybridized microwave-microfluidic sensor autonomously handled liquid samples to monitor the growth of *E. coli* over 24 hours and deduce the susceptibility of the pathogen against multiple antibiotics within 4 hours with a signal-to-noise ratio of 1107.5. This inexpensive and easily operated system shows a high potential to be implemented in POC environments while delivering high-throughput, accurate and reliable results, indicating an enormous improvement compared to contemporary gold-standard AST practices.

Authors thank Natural Sciences and Engineering Council of Canada (NSERC) for providing funding for this project.

A.S.N. thanks Canada Research Chair for providing funding for this project.

**References**

1. Centers for Disease Control U. Antibiotic Resistance Threats in the United States, 2019. [cited 2022 Jan 6]; Available from: http://dx.doi.org/10.15620/cdc:82532.

2. Feng J, Yee R, Zhang S, Tian L, Shi W, Zhang WH, et al. A Rapid Growth-Independent Antibiotic Resistance Detection Test by SYBR Green/Propidium Iodide Viability Assay. Front Med [Internet]. 2018 May 1 [cited 2022 Jan 11];5(MAY). Available from: https://pubmed.ncbi.nlm.nih.gov/29774213/

3. Khan ZA, Siddiqui MF, Park S. Progress in antibiotic susceptibility tests: a comparative review with special emphasis on microfluidic methods. Vol. 41, Biotechnology Letters. Springer Netherlands; 2019. p. 221–30.

4. WHO publisheD list of bacteria for which new antibiotics are urgently needed [Internet]. [cited 2020 Dec 10]. Available from: https://www.who.int/news/item/27-02-2017-who-publishes-list-of-bacteria-for-which-new-antibiotics-are-urgently-needed

5. Narang R, Mohammadi S, Ashani MM, Sadabadi H, Hejazi H, Zarifi MH, et al. Sensitive, Real-time and Non-Intrusive Detection of Concentration and Growth of Pathogenic Bacteria using Microfluidic-Microwave Ring Resonator Biosensor. Sci Rep [Internet]. 2018 Dec 25 [cited 2018 Nov 21];8(1):15807. Available from: http://www.nature.com/articles/s41598-018-34001-w

6. Mohammadi S, Narang R, Mohammadi Ashani M, Sadabadi H, Sanati‐Nezhad A, Zarifi MH. Real‐time monitoring of *Escherichia coli* concentration with planar microwave resonator sensor. Microw Opt Technol Lett [Internet]. 2019 Nov 25 [cited 2020 Apr 2];61(11):2534–9. Available from: https://onlinelibrary.wiley.com/doi/abs/10.1002/mop.31913

7. Safavieh R, Tamayol A, Juncker D. Serpentine and leading-edge capillary pumps for microfluidic capillary systems. Microfluid Nanofluidics. 2015 Jul 17;18(3):357–66.

8. Narang R. Developing a microfluidic-microwave platform for real-time, non-invasive and sensitive monitoring of pathogens and antibiotic susceptibility testing. 2020 Jun 25 [cited 2022 Jan 10]; Available from: https://prism.ucalgary.ca/handle/1880/113539

**Fig. 1 (abstract A4).**
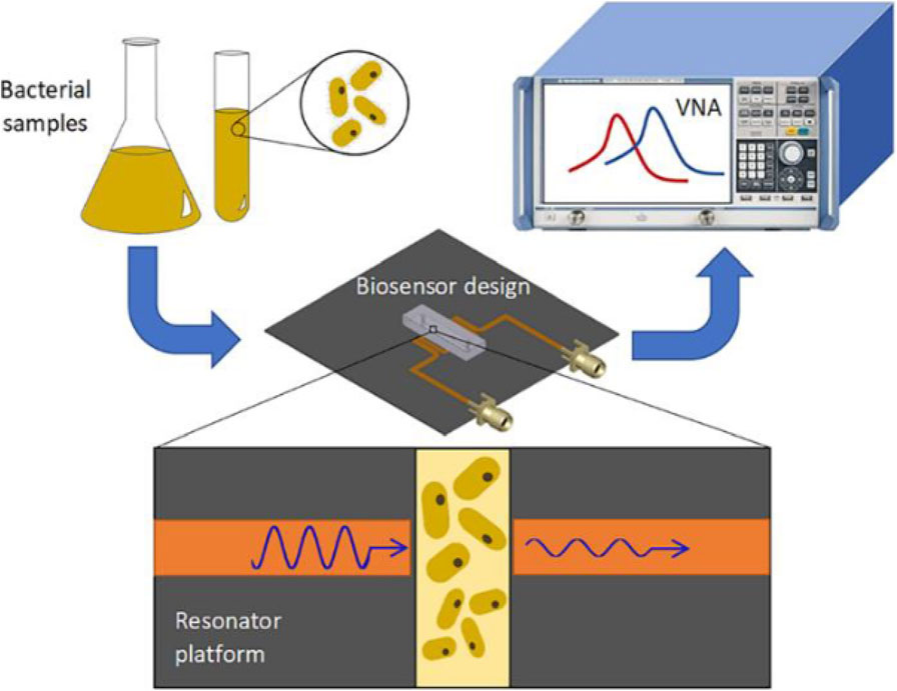
Schematic of the microwave-microfluidic sensor. The resonant profile is shifted due to the sample’s dielectric properties when the assay is introduced on the sensing region, analyzed through the vector network analyzer (VNA) ^[5]^

## A5. Cancer diagnosis using imaging and artificial intelligence applications

### Julia H. Miao, Kathleen H. Miao

#### Department of Biological Sciences, Cornell University, New York, NY, USA

Millions of people every year are affected by cancer globally. Through earlier diagnosis, cancer patient outcomes can be enhanced. Therefore, early detection of cancer is important for improving and saving lives. Artificial intelligence (AI) applications and cancer imaging are often utilized, such as increasing triage expediency in underserved regions worldwide, while aiding medical professionals[1,2].

**Materials and methods**

In this research, an AI model is developed using cancer imaging and machine learning with patient data to help increase cancer diagnosis and diagnostic accuracy[3]. The AI model was developed using AI algorithms to diagnose cancers in patients. Clinical patient data was applied to build, train, and test the model. For training, 50% of the patient data was randomly selected while the other 50% was used for testing its diagnosis abilities.

**Results**

In cancer diagnosis, the AI model achieved an overall 82% diagnostic accuracy compared to previously published methods ranging from 50% - 74% accuracy.

**Conclusions**

Therefore, AI algorithms and cancer imaging can be applied to aid healthcare professionals for diagnosing cancer in patients, such as in underserved regions worldwide in triage cases, improving outcomes and saving lives.

**References**

1. Sidey-Gibbons JAM, Sidey-Gibbons CJ. Machine learning in medicine: a practical introduction. BMC Med Res Methodol. 2019;19(1):64.

2. Shimizu H, Nakayama KI. Artificial intelligence in oncology. Cancer Sci. 2020;111(5):1452-1460.

3. Levine AB, Schlosser C, Grewal J, Coope R, Jones SJM, Yip S. Rise of the machines: advances in deep learning for cancer diagnosis. Trends Cancer. 2019;5(3):157-169

## A6. Enhancing the management of Long COVID in general practice: a scoping review

### Aimee Brennan^1^, John Broughan^1^, Geoff McCombe^1^, John Brennan^2^, Claire Collins^3^, Ronan Fawsitt^4,5^, Joe Gallagher^1^, Allys Guérandel^1,6^ , Brendan O’Kelly^1,7^, Diarmuid Quinlan^3^, John S Lambert^1,7^, Walter Cullen^1^

#### ^1^ School of Medicine, University College Dublin, Dublin, Ireland; ^2^ Royal College of Physicians of Ireland, Dublin, Ireland; ^3^ Irish College of General Practitioners, Dublin, Ireland; ^4^ Castle Gardens Surgery, Kilkenny, Ireland; ^5^ Ireland East Hospital Group, Dublin, Ireland; ^6^Department of Psychiatry and Mental Health Research, St Vincent’s University Hospital, Dublin, Ireland; ^7^ Mater Misericordiae University Hospital, Dublin, Ireland

**Background**

Long COVID is a multifaceted condition that has impacted a considerable proportion of those with acute-COVID-19 [1,2,3]. Affected patients often have complex care needs requiring holistic and multidisciplinary care, the kind routinely provided in general practice [4,5,6]. However, there is limited evidence regarding GP interventions. Aim This study aimed to address this issue by conducting a scoping review of literature on GP management of Long COVID. Method Arksey and O’Malley’s six-stage scoping review framework with recommendations by Levac et al. was used [7]. PubMed, Google Scholar, the Cochrane Library, SCOPUS, and Google searches were conducted to identify relevant peer-reviewed/grey literature, and the study selection process was conducted according to the PRISMA Extension for Scoping Reviews guidelines. Braun and Clarke’s ‘Thematic Analysis’ approach was used to interpret data. Results Nineteen of 972 identified papers were selected for review [8]. These included peer-reviewed articles and grey literature spanning a wide range of countries. Six themes were identified regarding GP management of Long COVID, these being: (i) GP uncertainty, (ii) Listening and empathy, (iii) Assessment and monitoring of symptoms, (iv) Coordinating access to appropriate services, (v) Facilitating provision of continual and integrated multi-disciplinary care and (vi) Need to facilitate psychological support. Conclusion The findings show that GPs can and have played a key role in the management of Long COVID, and that patient care can be improved through better understanding of patient experiences, standardised approaches for symptom identification/treatment, and facilitation of access to multidisciplinary specialist services when needed. Future research evaluating focused GP interventions is needed.

The authors would like to thank Ireland’s Health Research Board (HRB) funders of the North Dublin COVID-19 Cohort [ANTICIPATE] Study [COV19-2020-123], the family of the late Dr Mary J Farrell, the Ireland East Hospital Group, the UCD School of Medicine, and the UCD College of Health and Agricultural Sciences for supporting this project.

**References**

1. Aiyegbusi OL, Hughes SE, Turner G, et al. Symptoms, complications and management of long COVID: a review. J R Soc Med. 2021;114(9):428-42.doi: 10.1177/014107682110328502. Michelen M, Cheng V, Manoharan L, Elkheir N, Dagens D, Hastie C, et al. What are the long-term symptoms and complications of COVID-19: a protocol for a living systematic review. F1000Research. 2020;9:1455. doi: 10.12688/f1000research.27284.23. Tenforde MW, Kim SS, Lindsell CJ, Billig Rose E, Shapiro NI, Files DC, et al. Symptom Duration and Risk Factors for Delayed Return to Usual Health Among Outpatients with COVID-19 in a Multistate Health Care Systems Network - United States, March-June 2020. MMWR Morbidity and mortality weekly report. 2020;69(30):993-8. doi:10.15585/mmwr.mm6930e14. Berger Z, Assoumou S, Greenhalgh T. Long COVID and Health Inequities: The Role of Primary Care. Milbank Q. 2021; 99(2):519-541. doi: 10.1111/1468-0009.125055. Greenhalgh T, Knight M, Buxton M, Husain L. Management of post-acute covid-19 in primary care. Bmj. 2020;370. doi: 10.1136/bmj.m30266. Greenhalgh T, Knight M. Long COVID: A Primer for Family Physicians. Am Fam Physician. 2020;102(12):716-7. PMID: 333205117. Arksey H, O'Malley L. Scoping studies: towards a methodological framework. Int J of Soc Res Methodol. 2005;8(1):19-32. doi.org/10.1080/1364557032000119616 8. Braun V, Clarke V. Thematic analysis. Qual Res Psychol. 2012; 3(2) 77-101

## A7. The role of protein HuR in a glioblastoma cell under stress

### Miriam Matthews^1^, Yan Zhang^2^, Liang Lu^3^

#### ^1^University College Dublin, Dublin, Ireland; ^2^Baylor College of Medicine, Houston, Texas, USA; ^3^Michael E. DeBakey VAMC, Baylor College of Medicine, Houston, Texas, USA

##### **Correspondence:** Miriam Matthews (miriam.matthews@ucdconnect.ie)

**Background**

Protein HuR, an RNA binding protein has been identified as a key regulator of the proteins TDP-43 and FUS involved in the pathogenesis of ALS [1]. By understanding the role of HuR in a cell under stress, we hope to unravel the protective or pathogenic mechanisms that exist in a neuronal cell.

The objective of this study was to identify whether Protein HuR has a protective or detrimental effect on a neuronal glioblastoma cell under heat shock, toxic and oxidative stress.

**Methods**

shHuR (Protein HuR silenced) cells and shGFP (cells with protein HuR marked with green fluorescence protein) were used in this experiment to compare the cells with normal protein HuR and cells with a significantly smaller quantity of Protein HuR (Figure 1, Western blot analysis). After 24 hours of growth, both groups of cells were treated with either hydrogen peroxide or sodium arsenite added to the growth medium, or the plate was placed at 47°C incubation for two hours to generate heat shock. The cells were then counted by hand using a hemocytometer and the cell survival rate in the two groups were compared.

**Results**

Protein HuR has varying effects on cells in differing conditions of stress. In cells treated with hydrogen peroxide, shGFP had a statistically significant greater cell survival rate than shHuR (98% survival versus 28% survival), indicating that Protein HuR may have a protective effect on cells under oxidative stress. There was no observed difference in cell viability under conditions of 47°C heat shock. Both cells changed their morphology and detached compared to the control, but there was no statistically significant difference between cells from shGFP series and shHuR. The study with sodium arsenate indicated that Protein HuR had a protective effect on neuronal cells, as shGFP had a greater cell survival rate than shHuR.

**Conclusion**

The studies point to the protective effect of HuR for the survival of neuronal cells even when exposed to oxidative and toxic stress. There is no protective role for HuR in heat shock.

**Fig. 1 (abstract A7).**
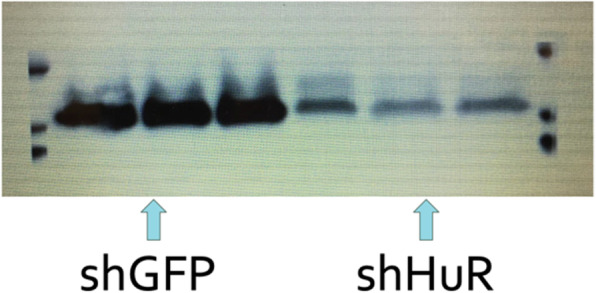
Western blot analysis demonstrating shGFP cells with Protein HuR and cells with silenced Protein HuR (shHuR)

**References**

1. Lu L, Zheng L, Si Y, et al. Hu antigen R (HuR) is a positive regulator of the RNA-binding proteins TDP-43 and FUS/TLS: implications for amyotrophic lateral sclerosis. J Biol Chem. 2014;289(46):31792-31804.

## A8. Feasibility of using a hand-held device to characterize tendon tissue biomechanics

### Sahand Sohirad^1^, David Wilson^1,2^, Charlotte Waugh^1^, Evan Finnamore^1,3^, Alexander Scott^1,3^

#### ^1^Centre for Hip Health and Mobility, Vancouver Coastal Health Research Institute, Vancouver, Canada, ^2^Department of Orthopaedics, Faculty of Medicine, University of British Columbia, Vancouver, Canada, ^3^Department of Physical Therapy, Faculty of Medicine, University of British Columbia, Vancouver, Canada

**Introductions**

The MyotonPRO (Myoton AS, Tallinn, Estonia) is a handheld, digital palpation device which has been used to measure the mechanical properties of muscles and other soft tissue. Using such a device, characterization of the biomechanical properties of the musculoskeletal system has the potential to help identify and diagnose abnormalities in skeletal tissues on-site and in the field without the need of other highly specialized equipment. For example, areas of increased muscle tone or the response of hypertonic muscle to therapeutic interventions have been reported. The MyotonPRO works by imparting a small mechanical impact to the tissue of interest, perpendicular to the surface of the skin. The tip of the probe is subjected to a constant preload to maintain contact and co-oscillation as the tissue vibrates underneath the skin. An accelerometer linked to the probe generates an acceleration vs. time relation from which various biomechanical characteristics, such as tissue stiffness, can be calculated.

The repeatability and reliability of the device have been tested on various muscles in several intra and inter-session studies, but never on tendon tissue to our knowledge. This study aims to conduct an independent assessment by a publicly funded, academic research group of the MyotonPRO’s stiffness measurement by (1) testing on phantom material with experimentally verified viscoelastic properties, (2) determining whether or not (and to what extent) the device’s measurements are influenced by the layer of skin over the tissue being measured, (3) examining the test-retest reliability of the MyotonPRO applied to human Achilles and patellar tendons and (4) examining the performance of the device during a field study of tendon stiffness in endurance runners.

**Methods:** The MyotonPRO was used to measure the stiffness and related properties of ballistics gel in comparison with an external materials testing system (PCB electronics). The device was then used to measure the same properties of avian Achilles tendons before and after the removal of the overlying skin and subcutaneous tissue. Next, the test-retest reliability of the Achilles and patellar tendons was determined in humans. Finally, the stiffness of the Achilles tendon was measured before and after competitive running races of varying distances (10, 21 and 42 km, total number of athletes analyzed = 66).

**Results:** The MyotonPRO demonstrated a high degree of consistency when testing ballistics gel with known viscoelastic properties. The presence of skin overlying the avian Achilles tendon had a statistically significant impact on stiffness (p<0.01) although this impact was of very small absolute magnitude (with skin; 728 Nm ±17 Nm, without skin; Nm 704 Nm ±7 Nm). In healthy adults of normal body mass index (BMI), the reliability of stiffness values was excellent both for the patellar tendon (ICC, 0.96) and the Achilles tendon (ICC,0.96). In the field study, men had stiffer tendons than women (p<0.05), and the stiffness of the Achilles tendon tended to increase following running (p = 0.052).

**Conclusions:** The MyotonPRO can reliably determine the transverse mechanical properties of tendon tissue. The measured values are influenced by the presence of overlying skin, however this does not appear to compromise the ability of the device to record physiologically and clinically relevant measurements.

## A9. Cross sectional study of wristband compliance in St Vincent’s University Hospital

### Patrick J Gorman^1,2^, Ian Callanan^2^

#### ^1^ School of Medicine, University College Dublin, Dublin, Ireland, ^2^ St Vincent’s University Hospital, Dublin, Ireland

##### **Correspondence:** Patrick J Gorman (Patrick.gorman@ucdconnect.ie)

**Background**

It is HSE policy that all patients should have a fit for purpose, laser printed, legible wrist- (or ankle-) band with a scannable barcode before receiving any care to avoid issues with misidentification ranging from the minor to the catastrophic. This re-audit aimed to show an improvement in the standard of compliance of wearing wristbands in the interest of patient safety.

**Methods**

498 patients were surveyed over a 7 day period to determine whether or not they had an identifying band on their person. In the instances that identification was missing follow up questions were asked as to who removed the band and why. Data was presented using Microsoft Excel.

**Results**

90.0% of patients had scannable ID bands on their person with the correct demographics (448/498). Patients themselves were primarily responsible for removing their own armbands with removal by a healthcare worker in the minority.

**Conclusions**

Compliance fell short of the ideal of 100%. The rate of compliance was at a similar level compared to previous audits carried out on the wearing of ID bands in SVUH, showing a lack of improvement and apathy for change. A further re-audit is not imminent due to the lack of institutional will.

## A10. Man vs machine: do mechanical chest compression devices improve survival outcomes in patients with out-of-hospital cardiac arrest – a systematic review

### Michael P. McElligott^1^, Dr. Alan Watts^2^

#### ^1^School of Medicine, University of Limerick, Limerick, Ireland; ^2^UHL Emergency Department, ALERT, Dooradoyle, Limerick, Ireland

##### **Correspondence:** Michael P. McElligott

**Background**

Mechanical chest compression devices (MCCDs) were developed to improve low survival rates from out-of-hospital cardiac arrest (OHCA)[1]. MCCDs have been shown to prevent practitioner fatigue, improve organisation, increase circulation and end-tidal CO_2_ pressures [2,3,4,5,6,7]. These benefits have not yielded an increase in survival outcomes. Previous systematic reviews show insufficient evidence to suggest superiority of MCCDs [8]. This review aimed to include new studies in order to answer the three-part question: “In [adults with OHCA], is the use of a [MCCDs better than manual CPR] at [achieving return of spontaneous circulation (ROSC) and/or improving survival rates]?”

**Materials and methods**

The keywords prehospital, out-of-hospital, paramedic, EMS, EMT, mechanical compression device/chest compressor, mechanical CPR, mCPR, Lucas, AutoPulse, survival and ROSC were searched in Embase, Pubmed, Ovid, Cochrane and ScienceDirect. Outcomes of interest included rates of ROSC, survival rates to admission, at 1 month/discharge, at 6 months and long term neurological function. Odds ratios (ORs) and 95% confidence intervals (CI) were utilised. Unreported CIs were obtained using the method outlined by Altmann and Bland[9]. The Levels of Evidence (LOE) assessment tool introduced by the International Liaison Committee on Resuscitation (ILCOR) 2010 was utilised in the evaluation and selection of literature. As a systematic review only, regression analysis or statistical tests for heterogeneity were not performed.

**Results**

Overall, 23 articles totalling 111,548 patients were included in the final analysis (PRISMA flowchart - Figure 1). The green, orange and red colour coding in the following Forest plots represent good, fair and poor quality of evidence respectively as outlined by the ILCOR LOE assessment tool.

Overall mean ORs after implementation of MCCDs showed no improvement in rates of achievement of ROSC/survival to admission, OR = 1.20 (95% CI, 0.86-1.56) (Figure 2); 1 month survival/survival to discharge, OR = 1.10 (95% CI, 0.71-1.56) (Figure 3); or long-term survival (6 months), OR = 1.18 (95% CI, 0.69-1.75) (Figure 4). Neurological outcomes, however, may be negatively impacted by MCCD use, OR = 0.75 (95% CI, 0.57-0.97) (Figure 5).

**Conclusions**

MCCDs appear to not effect survival rates of OHCA. They may, however, be associated with unfavourable neurological outcomes. The current recommendation that MCCDs can be used on a case-by-case basis when the practitioner predicts a benefit remains. Many studies identified certain sub-groups within OHCAs that have improved survival outcomes with MCCDs. As such, further large scale, randomised trials which focus on specific OHCA situations is required.

**Acknowledgments**

This is an abstract of the full-text study which was conducted as part of the assessment of final year medical students in the UHL School of Medicine.

A special thank you to Dr. Alan Watts whose guidance was paramount through this whole process and was always on hand with helpful and friendly advice.

**References**

1. Field JM, Hazinski MF, Sayre MR, Chameides L, Schexnayder SM, Hemphill R, et al. Part 1: Executive Summary: 2010 American Heart Association Guidelines for Cardiopulmonary Resuscitation and Emergency Cardiovascular Care.

2. Yuksen C, Prachanukool T, Aramvanitch K, Thongwichit N, Sawanyawisuth K, Sittichanbuncha Y. Is a mechanical-assist device better than manual chest compression? A randomized controlled trial. Open Access Emerg Med. 2017 Aug;Volume 9:63–7.

3. Rubertsson S, Karlsten R. Increased cortical cerebral blood flow with LUCAS; a new device for mechanical chest compressions compared to standard external compressions during experimental cardiopulmonary resuscitation. Resuscitation. 2005 Jun;65(3):357–63.

4. Steen S, Liao Q, Pierre L, Paskevicius A, Sjöberg T. Evaluation of LUCAS, a new device for automatic mechanical compression and active decompression resuscitation. Resuscitation. 2002 Dec;55(3):285–99.

5. Liao Q, Sjöberg T, Paskevicius A, Wohlfart B, Steen S. Manual versus mechanical cardiopulmonary resuscitation. An experimental study in pigs. BMC Cardiovasc Disord. 2010 Dec;10(1):53.

6. Duchateau F-X, Gueye P, Curac S, Tubach F, Broche C, Plaisance P, et al. Effect of the AutoPulse^TM^ automated band chest compression device on hemodynamics in out-of-hospital cardiac arrest resuscitation. Intensive Care Med. 2010 Jul;36(7):1256–60.

7. Axelsson C, Karlsson T, Axelsson ÅB, Herlitz J. Mechanical active compression–decompression cardiopulmonary resuscitation (ACD-CPR) versus manual CPR according to pressure of end tidal carbon dioxide (PETCO2) during CPR in out-of-hospital cardiac arrest (OHCA). Resuscitation. 2009 Oct;80(10):1099–103.

8. Zhu N, Chen Q, Jiang Z, Liao F, Kou B, Tang H, et al. A meta-analysis of the resuscitative effects of mechanical and manual chest compression in out-of-hospital cardiac arrest patients. Crit Care. 2019 Dec;23(1):100.

9. Altman DG, Bland JM. How to obtain the confidence interval from a P value. BMJ. 2011 Jul 16;343(aug08 1):d2090–d2090.

**Fig. 1 (abstract A10).**
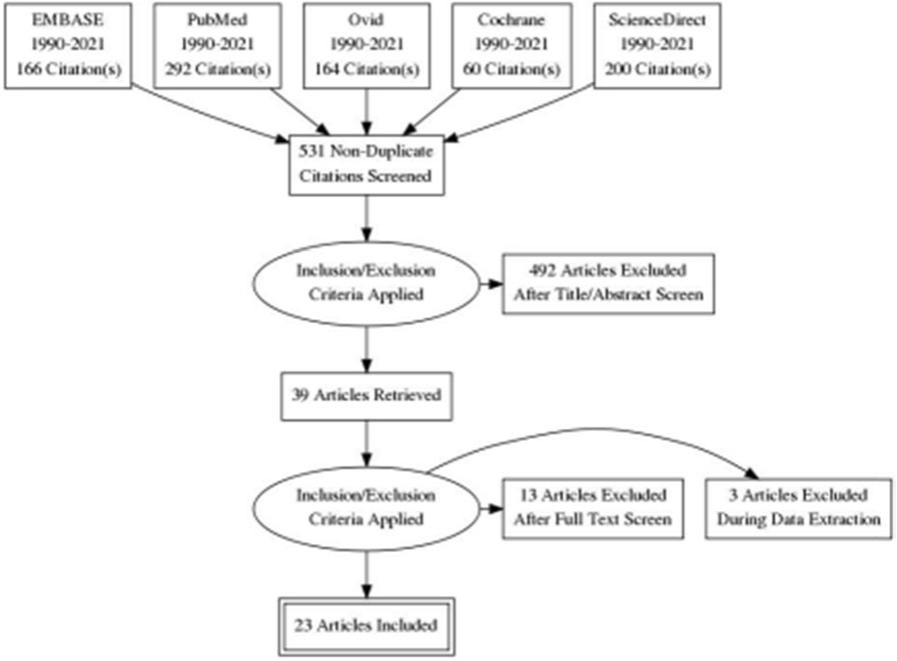
PRISMA flowchart outlining literature selection

**Fig. 2 (abstract A10).**
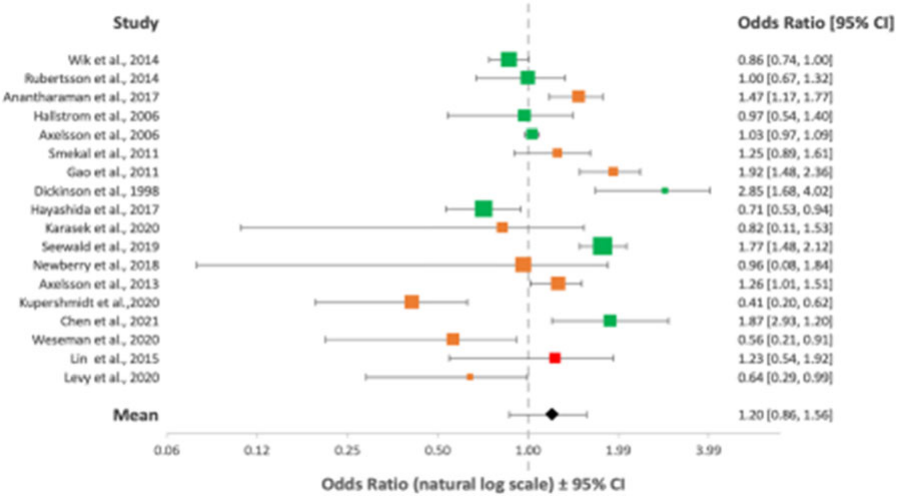
Forest plot displaying the studies comparing rates of short-term survival between manual CPR and MCCDs in OHCAs

**Fig. 3 (abstract A10).**
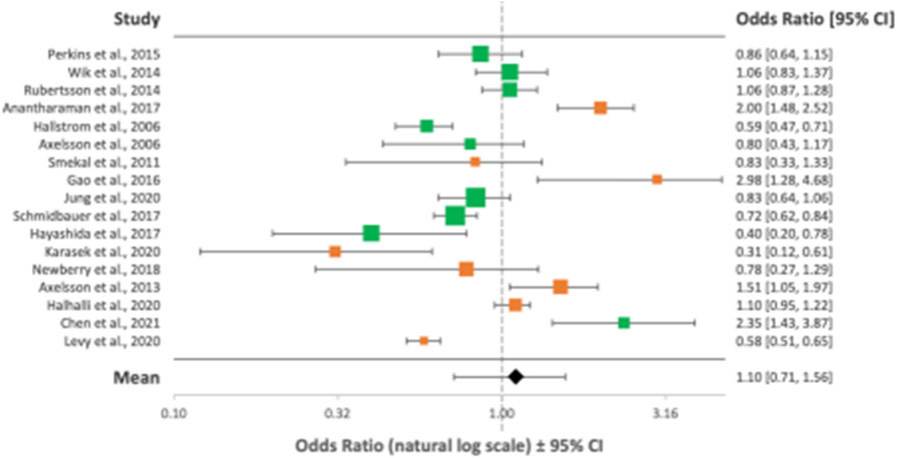
Forest plot displaying the studies comparing rates of medium term survival between manual CPR and MCCDs in OHCAs

**Fig. 4 (abstract A10).**
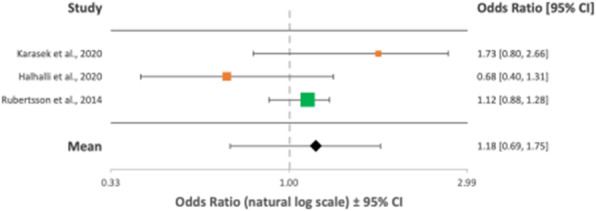
Forest plot displaying the studies comparing long-term survival between manual CPR and MCCDs in OHCAs

**Fig. 5 (abstract A10).**
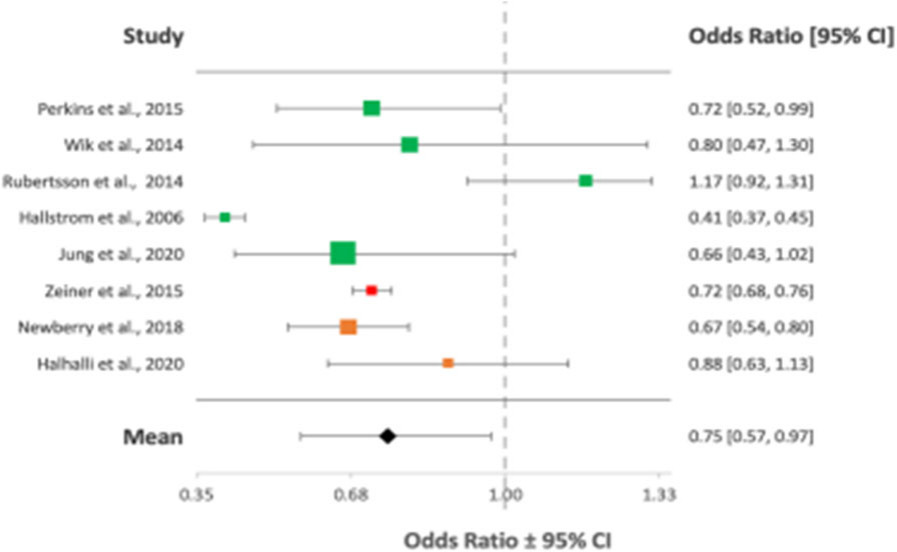
Forest plot displaying the studies comparing neurological outcomes between manual CPR and MCCDs in OHCAs

## A11. Analysis of anisometropia and amblyopia in the Republic of Ireland

### Aoife O’Donnell^1,2^, Aisling Roche^1^, Molly Thunder^1^, Michael Moore^1^

#### ^1^Technological University Dublin (TUD), School of Physics & Clinical & Optometric Sciences, Dublin Ireland; ^2^School of Medicine, Ulster University, Derry, Northern Ireland, United Kingdom

##### **Correspondence:** Aoife O’Donnell (aoifeodonnell@outlook.ie)

**Background**

The primary aim of this research project is to employ analytics on big data to investigate the levels of amblyopia and anisometropia in Ireland as well as the distribution of these conditions across the country. Comparing the prevalence of anisometropia and amblyopia in the Republic of Ireland (ROI) to that of other countries, according to year, refractive error and age act as the secondary aims.

**Methods**

Data analytics were retrospectively carried out on 143,234 unique patients across 296,797 visits. The gender division of the patients was 51.5% female, 34.3% male and was not recorded in 14.2% of cases. All 26 counties of the ROI were represented in the data although this was not evenly distributed throughout the country. This data was cleaned and analysed using the R statistical and the SQLite database programming languages. Statistical analysis and graphical representation were subsequently undertaken to produce and illustrate the results.

**Results**

The patients were aged between 0 and 100 years with an average age 47.5 ± 20.9 years. The estimated incidence of anisometropia and amblyopia were 15.8% and 4.2% respectively. Variables that did not impact upon prevalence levels for either of these disorders were county of residence and year of examination. Age and male gender were both weakly correlated with anisometropia. Meanwhile, increasing refractive error (hyperopic or myopic) had the strongest relationship with both prevalence levels. Although, the correlation was not significant enough with any of these variables to solely explain anisometropia development. In terms of amblyopia development, refractive error was the most influential variable with myopia being protective while anisometropia and hyperopia were strong predictive factors. However, hyperopia and anisometropia cannot exclusively account for the development of amblyopia.

**Conclusions**

This is the first study to provide population based data on the prevalence of anisometropia and amblyopia in the ROI. Although further research is required in this area, these results can be applied to improve the detection and management of these common and potentially inhibiting vision abnormalities.

This research was facilitated and supported by the TUD Optometry Department. The author would also like to acknowledge the participation of optical practices that provided data. The author is also grateful for the ongoing contribution and guidance of the project supervisor without whom this research project would not have been possible.

## A12. Investigating the necessity of pediatric emergency medicine in resource limited settings

### Avanti Baronia^1,2^

#### ^1^UCD School of Medicine, University College Dublin, Belfield, Dublin 4, Ireland; ^2^Arumeru District Hospital, Arusha, Tanzania

In Tanzania, the healthcare system is overburdened and lacking qualified healthcare workers. This is more pronounced in pediatrics. This study attempted to determine if a dedicated pediatric emergency care (PEC) program is possible in resource limited settings such as Tanzania.

The study was done through literature review of 13 papers and global recommendations on existing PEC standards and practices. The information gathered was then compared to field observations made in the Arumeru District Hospital of Arusha, Tanzania.

Though one third of patients seen in the Arumeru emergency department are children, staff are not trained to specifically care for pediatric cases. Additionally, the department lacks general emergency medicine resources, as well as equipment needed specifically for children.

The recommended personnel trained for pediatrics is 4.5 healthcare workers per 1,000 patients. Currently there are about 5 pediatricians per 100,000 patients in Tanzania [1]. Thus, the focus of resources and training should be to increase the number of healthcare workers and facilities able to handle pediatric cases in general, rather than focus on increasing the capacity for PEC specifically. Per World Health Organization (WHO) recommendations, rather than increasing specialized PEC practitioners (a long-term goal), resource limited settings can improve outcomes for pediatric emergencies in the short term by ensuring adherence to global emergency standards and training existing personnel in pediatric specific needs [2]. It is imperative that the standards be met at Arumeru Hospital before further consideration can be given to the necessity of developing designated pediatric emergency care programs.

**References**

1. Harper BD, Nganga W, Armstrong R*, et al.* Where are the paediatricians? An international survey to understand the global paediatric workforce. *BMJ Paediatrics Open.* 2019; **3:**bmjpo-2018-000397. doi: 10.1136/bmjpo-2018-000397

2. World Health Organization. Updated guideline: paediatric emergency triage, assessment and treatment: care of critically-ill children. World Health Organization. 2016; [cited: August 15^th^ 2021] https://apps.who.int/iris/handle/10665/204463

## A13. Robotic-assisted laparoscopic pyeloplasty for UPJ obstruction: The St. Michael’s Hospital experience

### Aren Mnatzakanian^1^, Melody Djuimo^2^, R. John D’A Honey^2, 4^, Jason Y. Lee^3, 4^ , Michael Ordon^2, 4^

#### ^1^UCD School of Medicine, University College Dublin, Belfield, Dublin, Ireland; ^2^Division of Urology, Department of Surgery, St. Michael’s Hospital, Toronto, Ontario, Canada; ^3^Division of Urology, Department of Surgery, University Health Network, Toronto, Ontario, Canada; ^4^Division of Urology, Department of Surgery, Temerty Faculty of Medicine, University of Toronto, Toronto, Ontario, Canada

**Background**

Ureteropelvic junction obstruction (UPJO) is a urologic condition that can cause flank pain, nephrolithiasis, recurrent urinary tract infections, and loss of ipsilateral renal function [1]. Robotic-assisted laparoscopic pyeloplasty (RAP) has been demonstrated to have a 90-95% success rate in treating UPJO [2]. At present, there is no literature on the outcomes of RAP in a Canadian context. Our objective was to perform a retrospective review of RAP cases at a high-volume Canadian centre.

**Methods**

We performed a retrospective chart review of patients that underwent RAP at St. Michael’s Hospital, between January 2012 and May 2019. Demographics, intra-operative details, and pre- and post-operative imaging results (ultrasounds, CT scans and renal Lasix scan (RLS)) were recorded. Patients were excluded if at least 1 year follow-up data was unavailable. Our primary outcome was clinical and radiologic improvement defined as (1) symptom improvement, (2) stable/improved split renal function on RLS and (3) either improvement in the degree of hydronephrosis on ultrasound or CT, or improved drainage time on RLS. Secondary outcomes included post-operative complications, need for diagnostic intervention (retrograde pyelogram or diagnostic ureteroscopy) and reintervention for recurrent UPJO.

**Results**

A total of 156 patients underwent RAP over the study time frame after exclusions. The median age was 42 and 66% were female (Table 1). Mean follow-up was 2.5 years. In terms of our primary outcome, 87% had clinical and radiologic improvement. Diagnostic investigation for possible recurrent/persistent obstruction, based on symptoms and/or imaging results, was required in 17% of cases, but only 3% required reintervention for recurrent UPJO. Accordingly, the overall treatment success was 97%. The most common post-operative complication was UTI (18%), and urine leak was seen in only 2% of patients.

**Conclusion**

The results of our retrospective review compare favourably with currently reported outcomes in the literature and demonstrate the safety and high level of success of RAP at a high-volume Canadian centre.

**References**

1. Al Aaraj MS, Badreldin AM. Ureteropelvic Junction Obstruction. StatPearls. Treasure Island (FL):    StatPearls Publishing Copyright © 2021, StatPearls Publishing LLC.; 2021.

2. Uhlig A, Uhlig J, Trojan L et al. Surgical approaches for treatment of ureteropelvic junction obstruction – a systematic review and network meta-analysis. BMC Urol. 2019; vol 19(1); 112.

**Table 1 (abstract A13).** Patient Demographics and Operative Details

Study Sample (N)	156
**Median Age at OR (IQR)**	**42 (28-58)**
Gender (%)	
M	**53 (34)**
**F**	**103 (66)**
**Mean BMI (SD)**	**25 (6)**
ASA status (%)	
I	**27 (17)**
II	**84 (54)**
III	**40 (27)**
**IV**	**3 (2)**
**Hypertension (%)**	**25 (16)**
**Type 2 Diabetes (%)**	**8 (5)**
**Prior History of Nephrolithiasis (%)**	**14 (9)**
**Prior Endoscopic Surgery (%)**	**19 (12)**
**Prior Endopyelotomy (%)**	**9 (6)**
**Side of Surgery (%)**	
**L**	**71 (46)**
**R**	**85 (54)**
**Mean OR Duration, min (SD)**	**178 (47)**
**Stones (%)**	**14 (9)**
**Crossing Vessel (%)**	**46 (30)**
**Mean Stent Duration, days (SD)**	**35 (7)**
**Mean LOS, nights (SD)**	**2 (1)**

## A14. Investigation of prescribing practices by Irish Veterinary practitioners

### Julie Edmondson, Carmel Mooney, Finola Leonard, Robert Shiel

#### UCD School of Veterinary Medicine, University College Dublin, Belfield, Dublin 4, Ireland

Antimicrobial resistance accession has resulted in scrutiny of both human and veterinary antimicrobial use. The purpose of the study was to investigate prescribing patterns of Irish small-animal veterinary practitioners. An anonymous online survey sent to registered veterinary surgeons collated demographic and prescribing data. Five hypothetical case scenarios were presented to explore antimicrobial use and the associated reasoning.140 clinicians participated in the survey, but only 82 (58.5%) completed all sections. 19 (13.6%) respondents reported that their practice had a policy for antimicrobial use. Veterinarians with 0-5, 6-15, 16-25 and ≥26 years’ experience prescribed antimicrobials in 28/70 (40.0%), 96/250 (38.4%), 82/230 (35.7%), and 53/140 (37.9%) scenarios, respectively. Duration qualified was not associated with the decision to prescribe antimicrobials (χ2(3, n=690)=0.61, p=0.89). The most common reasons influencing the decision to prescribe antimicrobials were in descending order: clinical signs, presumptive diagnosis, culture and susceptibility results, ease of administration, cytology results, financial constraints and client expectations. Amoxicillin-clavulanate was the most routinely prescribed antimicrobial in 99/274 (36%) of all prescriptions. This was underdosed in 17/86 (19.8%) prescriptions. Under general comments, 5/64 (7.8%) respondents described client expectations and pressures influencing prescribing practices, 5/64 (7.8%) indicated that expense influenced the ability to perform culture and susceptibility testing, and 5/64 (7.8%) described the use of empirical prescribing. This study demonstrated that antimicrobial use is not influenced by the number of years in practice but is influenced by several clinical and owner-dependent factors. Amoxicillin-clavulanate a European Medicines Agency Class C antimicrobial, was widely used and frequently at doses less than commonly accepted guidelines.

Acknowledgements to Professor Carmel Mooney, Finola Leonard and Robert Shiel for their ongoing support and help with the research. Also many thanks to my research partner Carli Gentile.

## A15. Analysis of stakeholder perception of comparative oncology in the study of melanoma

### Bridget Collins ^1^, Stephanie Bollard^1,2,3^, Jane Howard^1,2^, Clodagh Canavan^3^, Pamela Kelly^4^, Amanda McCann^1,2^, Shirley Potter ^1,2,3^

#### ^1^UCD School of Medicine, University College Dublin, Belfield, Dublin 4, Ireland; ^2^UCD Conway Institute of Biomedical and Biomolecular Research, University College Dublin, Belfield, Dublin 4, Ireland; ^3^Department of Plastic, Reconstructive & Aesthetic Surgery, Mater Misericordiae University Hospital, Phibsborough, Dublin 7, Ireland; ^4^UCD School of Veterinary Medicine, University College Dublin, Belfield, Dublin 4, Ireland

Comparative oncology examines naturally occurring cancers seen in both animals and humans to compare findings between species. It is a growing field which has the potential to benefit both veterinary and human patients by giving insights into cancer progression and treatment responses. Importantly, comparative oncology requires collaboration between many groups including Veterinary Professionals, Human Healthcare Professionals, Biomedical Researchers, Pet Owners, and People with Lived Experience of Cancer. Our study aimed to qualitatively assess the different perceptions and knowledge of comparative oncology between these various stakeholders.

Interviews and a survey were conducted by senior researchers analysing the perceptions of various stakeholder groups involved in Comparative Oncology: Veterinarians, Patients, Healthcare Professionals and Biomedical Researchers. Information was included on Respondent Subgroups, Pet Ownership status, as well as opinions on Communication of Findings, Consent, Knowledge, Opinions, and Values/Concerns. These interviews and surveys were analysed in NVivo using matrix coding and standardised in Excel using a mentions per person ratio to assess perceptions across subgroups.

176 individuals responded to the anonymous survey, and a further 12 individuals were interviewed to assess their knowledge and perceptions regarding Comparative Oncology. The stakeholder groups presented with various levels of knowledge and concerns regarding Comparative Oncology. Expectedly, the biomedical researcher cohort had the greatest knowledge mentions per person of 1.9, followed closely by Veterinary professionals (1.4), with the lowest being Human Healthcare Professionals (0.55). The Concerns held by respondents were classified as “Animal Welfare”, “Convenience”, “Cooperation with Veterinary Professionals”, “Data Management/Storage”, “Information Availability”, “Scientific Rigour”, and “No Concerns”.

In our study, the stakeholder groups held different perceptions of comparative oncology. Researchers were most concerned with scientific rigour whilst Veterinarians were most often concerned with animal welfare.

The results of this study indicate the different values held by the stakeholders in comparative oncology and indicate areas researchers must address when undertaking these studies.

## A16. Improving infection control in the UCD Veterinary Hospital: sources and persistence of faecal contamination

### Ashokkumar Singaravelu^1^, Bernadette Leggett^2^, Finola Leonard^2^

#### ^1^UCD School of Medicine, University College Dublin, Belfield, Dublin 4, Ireland; ^2^UCD School of Veterinary Medicine, University College Dublin, Belfield, Dublin 4, Ireland

##### **Correspondence:** Ashokkumar Singaravelu (ashokkumar.singaravelu@ucdconnect.ie)

**Background**

Enterococci and *Escherichia coli* can survive on inanimate surfaces for months and pose a threat to human and animal health [1]. The UCD Veterinary Hospital (UCDVH) is screened every year or when there is a multi-drug resistant (MDR) case on site. A longitudinal study was conducted to intensely monitor sites that were contaminated with antimicrobial-resistant faecal bacteria for 3 weeks. The main purpose of this study was to evaluate cleanliness and microbial burden in the UCDVH to establish the extent of cross-contamination with faecal bacteria as part of a review of infection control procedures within the hospital to aid in reducing nosocomial infections.

**Materials and methods**

Environmental surfaces, and hand hygiene were assessed using 3M^TM^ Clean-Trace^TM^ ATP test, 3M^TM^ Petrifilm^TM^ plates, bacteriological culture of *Enterococcus* species and *E. coli* and antimicrobial susceptibility testing as appropriate. A cross-sectional study was conducted to identify sources of contamination within the hospital, followed by a longitudinal study to identify the persistence of bacteria. Records of in-patients hospitalised during the 8-week study period and from which either *E. coli* or enterococci had been isolated were reviewed to determine the relationship with environmental contamination and possible hospital-acquired infections. Cross-contamination was assessed through antimicrobial resistance typing.

**Results**

Of 113 samples from the cross-sectional study, *Enterococcus* species were isolated from 31 (27.4%) and *E. coli* from 9 (7.9%). Four of 51 (7.8%) of hand samples were contaminated with these pathogens. Twenty-one isolates (28.8%) were MDR (Figure 1). There was no change in cleanliness or microbial burden over 3 weeks. Enterococci and *E. coli* isolates with same resistance patterns were recovered from the environment in the large and small animal hospitals and from a small number of patients (Figures 1, 2).

**Results**

These results suggest that movement between the small and large animal hospital areas may be responsible for cross-contamination and possible hospital-acquired infections. These data will inform an imminent review of infection control protocols and hygiene procedures by the UCDVH Infection Control Committee.

**Acknowledgements**

The study was supported by the University College Dublin Veterinary Hospital. We thank all staff members and students for their cooperation. We thank Seán Lacey for his help in conducting the statistical analyses in R Studio. We also thank Justin Lyng, Dr George Cloughley and Dr Mary Sekiya for their support during the study.

**References**

1. Kramer A, Assadian O. Survival of Microorganisms on Inanimate Surfaces. In: Borkow G. Use of Biocidal Surfaces for Reduction of Healthcare Acquired Infections. Springer, Cham; 2014. p.7-26.

**Fig. 1 (abstract A16):**
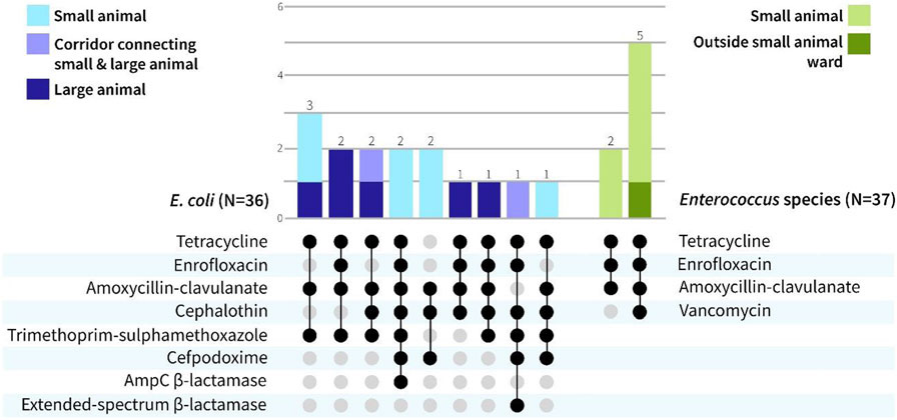
Observed antimicrobial resistance combinations for MDR *E. coli* (left) and MDR *Enterococcus* species (right) and the frequency of these combinations (top). MDR bacteria were defined as resistant to at least 3 classes of antimicrobials

**Fig. 2 (abstract A16):**
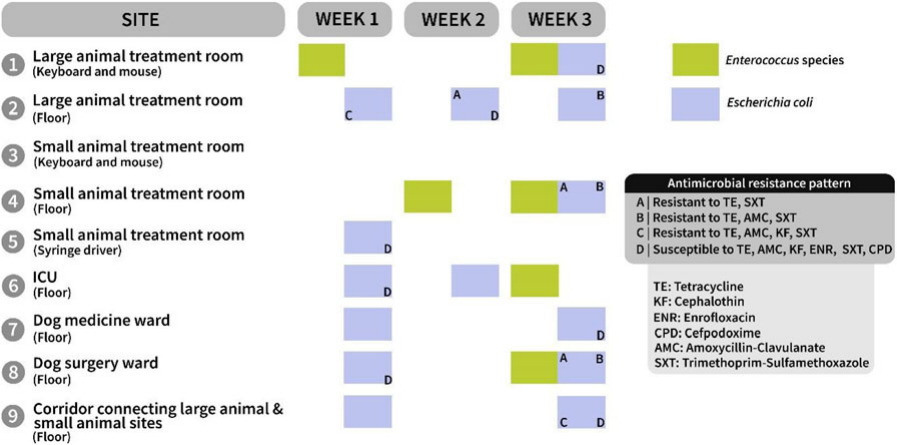
Isolates of *Enterococcus* species (in green) and *E. coli* (in lavender-blue) with the same antimicrobial resistance pattern (A-D) were recovered from different sites

## A17. Why is it so hard to get adolescent feedback? A report on a short-term feedback project in a paediatric hospital

### Alexa L Mitroi^1^, Elizabeth Barrett^1,2^, Siobhan Sheehy^2^, John Butler^2^, Carole Boylan^2^

#### ^1^UCD School of Medicine, University College Dublin, Belfield, Dublin 4, Ireland; ^2^Children’s University Hospital, Temple Street, Dublin 1, Ireland

**Background**

Children are capable of reflecting critically upon the services they receive, making them competent consumers of mental health services [1]. Examining children’s feedback gives direct insight into what is working and where further improvement is needed in the way care is delivered [2]. This project aimed to provide feedback to the mental health team about the experiences of patients at Children’s University Hospital (CUH), considering their impression of the environment and meeting the mental health team for the first time.

**Methods**

Ethical exemption was granted by the hospital ethical committee. An initial literature search on child and carer feedback for paediatric consultation-liaison psychiatry services was conducted using PubMed. 42 articles were identified based on title and/or abstract, and 25 articles were selected based on relevance to the topic. Articles were analysed qualitatively using thematic analysis. To collect feedback, a questionnaire with opt-in Likert scale and free text questions was given to 20 children and their carers to be completed anonymously. Four questionnaire sets were returned to the team over a four-week period. Questionnaire data were analyzed using Excel and qualitative analysis.

**Results**

Two main themes were identified in the literature: ‘Impact of child and adolescent voices in psychiatric services’ and ‘Addressing the concerns and expectations of children and adolescents’. Evidence suggests that patient feedback tools can improve treatment engagement and may improve patient outcomes in child and adolescent mental health services. Literature supports addressing the expectations and concerns of children and adolescents to increase patient satisfaction and improve the overall quality of mental health services. Participating patients were female adolescents (n=4) and their carers (n=4). Adolescents were least satisfied with the physical environment of the hospital and most satisfied with the recreational activities offered and the extent to which the team listened to them. Adolescents shared both positive and negative experiences and provided tangible feedback on the environment and care. Carers were satisfied with their own experience and their child’s experience.

**Conclusions**

Literature review findings reinforce the importance of patient feedback for child and adolescent mental health services. Child and carer feedback should continue to be collected to help improve the experience of patients at CUH. However, it was difficult to collect feedback from adolescents. To increase response rates, open-ended questions should be optional, and a digital questionnaire format should be made available [3].Questionnaire clinical utility and consumer appeal should also be taken into consideration [4].

**References**

1. Biering P. Child and adolescent experience of and satisfaction with psychiatric care: a critical review of the research literature. Journal of Psychiatric and Mental Health Nursing. 2010;17(1):65-72.

2. Hardavella G, Aamli-Gaagnat A, Saad N, Rousalova I, Sreter KB. How to give and receive feedback effectively. Breathe (Sheff). 2017;13(4):327-33.

3. Response rate [Internet]. SurveyMonkey Help Center. [cited 2021Jul24]. Available from: https://help.surveymonkey.com/articles/en_US/kb/Response-Rate-Tips-How-to-improve-low-response-rates

4. Kelley SD, Bickman L. Beyond outcomes monitoring: measurement feedback systems in child and adolescent clinical practice. Curr Opin Psychiatry. 2009;22(4):363-8.

## A18. Biosensors for cardiovascular mechanical circulatory support devices: a literature review

### Muhammad Zain Ali^1^, Mahmoud Abbassy^1^, Aamir Hameed^2^

#### ^1^School of Medicine, RCSI University of Medicine and Health Sciences, Dublin 2 Dublin, Ireland; ^2^Tissue Engineering Research Group (TERG), Department of Anatomy and Regenerative Medicine, RCSI University of Medicine and Health Sciences, Dublin 2 Dublin, Ireland

**Background**

Heart failure continues to be a leading cause of mortality worldwide [1]. Different markers and changes within the body can allude to different cardiac pathologies. These changes can be detected by specific biosensors [2]. Coupling or implanting these biosensors with mechanical circulatory support devices (MCSDs), such as left ventricular assist devices (LVADs), can be extremely important in detecting changes in biochemical and physiological parameters following MCSD implantation [3]. The aim of this review is to explore the available biosensors that may be coupled or implanted alongside LVADs to monitor biomarkers, such as interleukin 10 (IL-10), and changes in physiological parameters, such as ventricular pressure. This review will also explore the potential for feedback control mechanisms to be integrated with LVADs in response to the detected parameters. An exploration of the different materials used to fabricate biosensors is also presented.

**Methods**

We searched PubMed and Web of Science databases. Keywords included: biosensors, LVADS, MCSDs, sensors, and heart failure. Studies were included if they mentioned the testing of a biosensor detecting any biochemical or physiological parameter and the device was implanted on or alongside a MCSD.

**Results**

Of the 488 results obtained, a total of six studies met the inclusion criteria. A range of in-vivo and in-vitro studies were selected for this review. Two studies aimed to detect biochemical parameters in-vitro and successfully detected interleukin-10 and tumour necrosis factor-α when implanted alongside LVADs [4,5]. A total of four studies, two of which tested in-vitro and the remaining two tested in-vivo, aimed to detect physiological parameters and successfully detected changes in blood pressure [6,7,8,9]. Two studies also offered mechanisms for feedback control of the MCSD based on pressure input [7,9]. Regarding fabrication of these biosensors, the materials most used to fabricate biosensors are synthetic polymers, metals, carbon-based, and glass/silicon. Of these materials, synthetic polymers are the most common [10].

**Conclusions**

Implanting biosensors alongside MCSDs has the potential to improve patient outcomes and identify pathologies before they arise. The existing research offers promising results because as MCSDs become increasingly popular, there is potential to develop and integrate these biosensors to better meet our needs for rapid diagnostic and prognostic real-time information. Future research should be aimed at testing these devices in-vivo as well as developing feedback control mechanisms.

**References**

1. Lippi G, Sanchis-Gomar F. Global epidemiology and future trends of heart failure. AME Medical Journal. 2020;5:15-15.

2. Gray M, Meehan J, Ward C, Langdon S, Kunkler I, Murray A et al. Implantable biosensors and their contribution to the future of precision medicine. The Veterinary Journal. 2018;239:21-29.

3. Horvath D, Karimov J, Byram N, Kuban B, Sunagawa G, Moazami N et al. Advantages of Integrating Pressure-Regulating Devices Into Mechanical Circulatory Support Pumps. ASAIO Journal. 2019;65(1):1- 3.

4. Baraket A, Lee M, Zine N, Sigaud M, Bausells J, Errachid A. A fully integrated electrochemical biosensor platform fabrication process for cytokines detection. Biosensors and Bioelectronics. 2017;93:170-175.

5. Baraket A, Lee M, Zine N, Yaakoubi N, Trivella M, Elaissari A et al. A Flexible Label-Free Biosensor Sensitive and Selective to TNF-α: Application for Chronic Heart Failure. Sensors & Transducers. 2014;27:15-21.

6. Hubbert L, Baranowski J, Delshad B, Ahn H. Left Atrial Pressure Monitoring With an Implantable Wireless Pressure Sensor After Implantation of a Left Ventricular Assist Device. ASAIO Journal. 2017;63(5):60-65.

7. Fritz B, Cysyk J, Newswanger R, Weiss W, Rosenberg G. Development of an Inlet Pressure Sensor for Control in a Left Ventricular Assist Device. ASAIO Journal. 2010;56(3):180-185.

8. Zhou M, Yang C, Liu Z, Cysyk J, Zheng S. An implantable Fabry-Pérot pressure sensor fabricated on left ventricular assist device for heart failure. Biomedical Microdevices. 2011;14(1):235-245. 9. Qawasma R, Daud A. Left Ventricular Heart Assist Device using MC and ECG Feedback. International Journal of Biosensors & Bioelectronics. 2017;3(3).

10. Xu M, Obodo D, Yadavalli V. The design, fabrication, and applications of flexible biosensing devices. Biosensors and Bioelectronics. 2019;124-125:96-114.

## A19. Likely multisystem inflammatory syndrome in an adult following the mRNA SARS-CoV-2 vaccine

### Harry Kearns^1^, Ross Murphy^2^, Deirdre O’Donovan^2^

#### ^1^UCD School of Medicine, University College Dublin, Belfield, Dublin 4, Ireland; ^2^Blackrock Clinic, Co. Dublin, Ireland

Although myocarditis is now a well-known but rare complication of mRNA SARS-CoV-2 vaccine, multisystem inflammatory syndrome (MIS) is an extremely rare complication. This case details a likely MIS in a 21 year old male who presented 20 days after a second dose of the mRNA Pfizer-BioNTech COVID-19 vaccine. He had no prior history of SARS-CoV-2 infection but his history was significant for anaphylaxis to egg and chicken. He presented with a two day history of progressive retrosternal odynophagia, mild dull chest pain which was exacerbated by lying flat and deep inspiration and lethargy. Laboratory investigations revealed a markedly elevated troponin level at 1061 ng/L (normal <34) and CRP was raised at 20.8 mg/l (normal 5.0). ECG showed normal sinus rhythm, ST elevation in leads V4 and V5 and T wave inversion in V5.

On admission, symptoms worsened and he was unable to lie flat and eat food or drink liquids. He was commenced on diclofenac acid and a proton pump inhibitor. Echocardiogram and cardiac MRI were normal and oesophagogastroduodenoscopy (OGD) showed severe ulcerative oesophagitis with sloughing from the oesophagogastric junction to 32cm ab oral. Following the OGD, diclofenac was discontinued and colchicine introduced. PICC line was inserted on day 6 and total parenteral nutrition was commenced. Further tests showed raised LFTs and telemetry showed several short bursts of ventricular tachycardia during the two-week hospital stay so a beta-blocker was introduced.

The patient slowly recovered and repeat OGD four months later showed complete recovery of the ulceration but histopathology showed likely eosinophilic oesophagitis. Of note, the patient had no previous symptoms of this disorder and remains well in this regard and has fully recovered. A repeat OGD is planned. This interesting case of likely MIS raises the possibility that this vaccine response may be more likely to occur in those with an allergic history.

## A20. Online reflective practice groups for interdisciplinary trainees in paediatric hospitals during Covid-19 pandemic: what’s the evidence?

### Dwarika Raveena^1^, Tiedt Iolanda^2*^, Barrett Elizabeth^2^

#### ^1^UCD School of Medicine, University College Dublin, Belfield, Dublin, Ireland; ^2^Child and Adolescent Psychiatry, Temple Street Children’s University Hospital, Temple Street, Dublin, Ireland

In 2017, the report on the National Wellbeing of Doctors proposed that there are ever-increasing burnout rates [1]. Reflective practice groups are used to explore a deep level of understanding of doctor-patient relationships, in order to combat burnout and increase satisfaction at work[2]. This study aims to assess online reflective practice groups for interdisciplinary trainees in Paediatric hospitals during the Covid-19 pandemic.

The Balint group methodology was adapted for an online format. Trainees from psychiatry, emergency and paediatric specialties answered two online questionnaires before and after six sessions of Balint group meetings. There were nine responses to the pre-Balint questionnaire and eight responses to the post-Balint questionnaire. The data was analysed using Microsoft Excel.

75% of participants were from Crumlin Children’s Hospital. Most were women, aged 26-30 years and 3-11 years’ experience. Six participants preferred online groups while four preferred face to face groups after the sessions were completed. Trainees indicated that they thought about patient cases afterward and their teams were disrupted which may cause mild burnout due to the struggles faced. There was a positive relation between burnout reduction and Balint sessions. Additionally, the sessions were positively reviewed by the trainees and there were sessions cancelled which may indicate the trainees’ appreciation for the group.

Reflective practice programs should be implemented for trainees in all institutions since there is a positive link between reducing the risk of burnout and reflective practice groups. It should be available for all specialties, not only psychiatry and general practice.

References:

1. Kehoe C, Barrett E. Doctor’s burnout and interventions*. Irish Journal of Psychological Medicine*.2020:1-3.

2. Diskin C, Russell R, Barrett E. *The introduction of reflective practice at Temple Street- an experience with a NCHD Balint Groups [PDF]*. Irish Network of Healthcare Educators: Temple Street Children’s University Hospital; 2018.

## A21. Practices for identifying mental health issues in elite athletes: A scoping review

### A O’Keeffe^1^, J Broughan^1^, G McCombe^1^, A Lane^1^, G Fitzpatrick^2^, M Roe^3^, T Crowley^1^, W Cullen^1^

#### ^1^School of Medicine, University College Dublin, Ireland; ^2^Department of Health, Sport, and Exercise Science, Waterford Institute of Technology, Waterford, Ireland; ^3^School of Public Health, Physiotherapy and Sport Science, University College Dublin, Ireland

**Background**

Elite athlete mental health is becoming a topic of increasing interest. Following the publication of the International Olympic Committee consensus statement [1] in 2019, emphasis has been placed on encouraging help-seeking and treatment for elite athletes with mental health issues. However, there remains a paucity of research into the diagnostic practices and screening tools that exist to aid in identifying mental ill health in elite athletes. This study aims to examine the question “What identification practices exist to aid in identifying mental ill health in elite athletes?”

**Methods**

A scoping review design was undertaken following the six-stage process developed by Arksey and O’Malley [2] with revisions by Levac et al [3]. The PubMed, SPORTDiscus and PsycINFO databases were searched for relevant papers. This review follows in accordance with the Preferred Reporting Items for Systematic Reviews and Meta-Analyses extension for Scoping Reviews. (PRISMA-ScR).

**Results**

Forty-three studies were included in the review. Emerging themes concerned the importance of timely identification, the need to make identification pathways/resources accessible, the delivery of interventions, and using the right tools for identification. A range of mental health outcome measures were identified, few of which were athlete specific.

**Conclusion**

Practices for identifying mental ill-health in elite athletes are numerous and varied. Many are of questionable use in elite athlete populations, and few screening tools are specific to elite athletes. Many countries and sport organisations lack consensus-based guidelines for identifying mental health problems in elite athletes. Further research to develop high-quality athlete-specific screening tools should be a priority.

**References**

1. Reardon, C.L., et al., Mental health in elite athletes: International Olympic Committee consensus statement (2019). British Journal of Sports Medicine, 2019. 53(11): p. 667.

2. Arksey, H. and L. O'Malley, Scoping studies: towards a methodological framework. International journal of social research methodology, 2005. 8(1): p. 19-32.

3. Levac, D., H. Colquhoun, and K.K. O'Brien, Scoping studies: advancing the methodology. Implementation Science, 2010. 5(1): p. 69.

## A22. Are plastic surgery trainees accurate assessors of their own microsurgical skill?

### David Carolan^1^, Robert Milling^2^, Christine Quinlan^2^, Shirley Potter^1,2^

#### ^1^UCD School of Medicine, University College Dublin, Belfield, Dublin 4, Ireland; ^2^Dept of Plastic and Reconstructive Surgery, Mater Misericordiae University Hospital, Eccles St, Dublin 7, Ireland

**Background**

Microsurgery is a highly skilful component of Plastic and Reconstructive surgery with a steep learning curve. Due to COVID-19, reduced access to technical courses and hands-on theatre time has created significant challenges in microsurgical education . Trainees must therefore engage in self-education and be adept at accurate self-assessment to overcome this. The aim of this study was to assess the ability of trainees to self-assess technical performance while performing a simulated microvascular anastomosis.

**Materials and Methods**

Novice and experienced Plastic surgery trainees were recruited. All participants performed a simulated microvascular anastomosis by placing 8 interrupted, evenly spaced sutures around a high fidelity chicken femoral vessel model. A stopwatch timed the procedure from start to finish. Each participant objectively rated their anastomosis using the Anastomosis Lapse Index (ALI) [1]. Each anastomosis was then blindly rated by two expert microsurgeons. Self-scores and expert scores were compared using a Wilcoxon-Signed Rank Test.

**Results**

Thirteen surgical trainees completed the simulated procedure. Mean time to completion was 22.2 minutes (range 14.2-31.9 minutes). Mean ALI self-score was 3.8 (range 3-5) while mean ALI expert score was 5.27 (range 4.5-6). There was a significant difference between ALI self-score and expert score (p=0.001) with expert assessors consistently assigning a higher ALI score to the same anastomosis (Figure 1). There was no significant difference between male and female trainees or between novice and experienced trainees in relation to time to completion, ALI self-score or ALI expert score.

**Conclusions**

These findings suggest that while the ALI is an excellent training tool, surgical trainees tend to overestimate their technical performance. This emphasises the importance of expert feedback to accurately self-assess progress in the early stages of surgical training.

**References:**

1. Ghanem, A. M., Al Omran, Y., Shatta, B., Kim, E., & Myers, S. Anastomosis Lapse Index (ALI): A Validated End Product Assessment Tool for Simulation Microsurgery Training. *Journal of reconstructive microsurgery. 2016*, *32*(3), 233–241.

**Fig. 1 (abstract A22).**
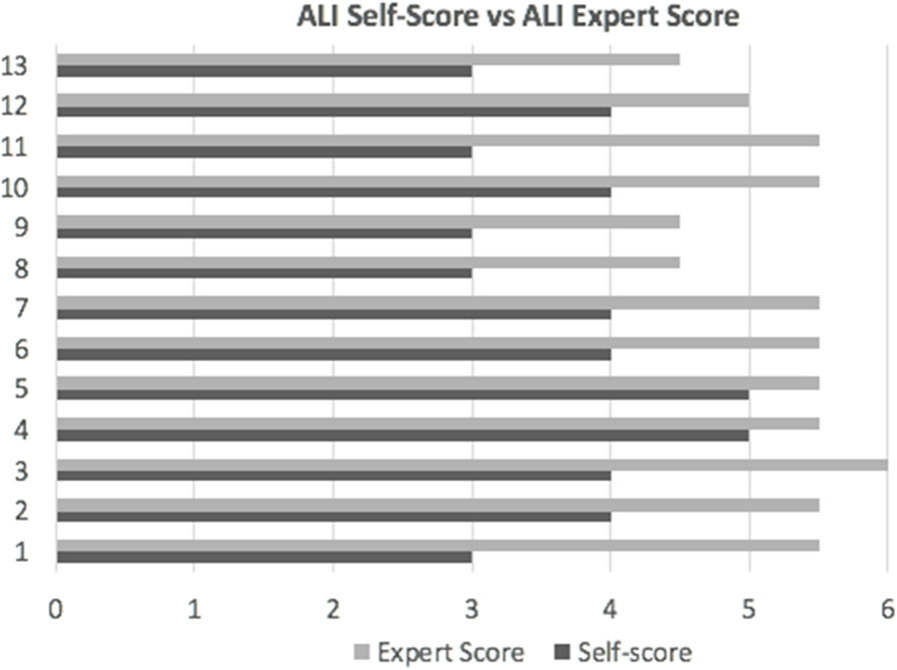
ALI Self-Score vs ALI Expert Score

## A23. A prospective audit of the clinical implementation of urine neutrophil gelatinase-associated lipocalin (uNGAL) as a biomarker of acute kidney injury in hospitalised patients

### Therese Meade^1^, Jean Maxime Côté^1,2,3^, Liam Lyons^1,4^, Patrick J Twomey^1,4^, Ted J Fitzgerald^1,5^, Jia Wei Teh^1,5^, John Holian^1,5^, Aisling O’Riordan^1,5^, Alan Watson^1,5^, Michelle Clince^5^, Fahad Malik^5^, John O’Regan^1,5^, Patrick T Murray^1,2,5^

#### ^1^ UCD School of Medicine, University College Dublin, Belfield, Dublin 4, Ireland; ^2^ Clinical Research Centre, University College Dublin, Belfield, Dublin 4, Ireland; ^3^ Division of Nephrology, Centre Hospitalier de l’Université de Montréal, Montréal, Canada; ^4^ Department of Clinical Chemistry, St. Vincent’s University Hospital, Elm Park, Dublin 4, Ireland; ^5^ Department of Nephrology, St. Vincent’s University Hospital, Elm Park, Dublin 4, Ireland

**Background**

Novel acute kidney injury (AKI) biomarkers have been shown to improve diagnostic accuracy, but reports of use in standard clinical practice are rare. The objective of this audit is to evaluate the clinical utility of urine Neutrophil gelatinase-associated lipocalin (uNGAL) in newly diagnosed AKI episodes in hospitalized patients.

**Materials and Methods**

We reported the implementation of uNGAL measurement for routine AKI diagnostic workup of patients receiving nephrology consultation in an academic centre, focusing on discrimination of AKI aetiology (functional/pre-renal vs intra-renal), using retrospective data collection. The diagnostic accuracy of uNGAL was compared to the final adjudication by two independent nephrologists, using descriptive statistics (Mann-Whitney U test) and Area Under The Receiver Operating Characteristic Curve (AUC- ROC) analysis.

**Results**

Results from 55 uNGAL samples were added to the original cohort (46 samples) previously audited, in a hospital-approved audit extension. KDIGO stage 1, 2, 3 AKI and renal replacement therapy occurred in 7%, 36%, 57% and 23% of cases, respectively. The median uNGAL level was 107.6 (46.3-357.9) ng/mL for functional/pre-renal AKI and 930.0 (239.0-1296.1) ng/mL for intra-renal AKI (p=<0.001). In patients without UTI, median uNGAL was 84.5 (41.5-315.0) ng/mL for pre-renal and 908.9 (223.6-1360.5) ng/mL for intra-renal (p=<0.001). Median uNGAL/uCreatinine in cases without UTI was 1.23 (0.71-4.50) ng/mg in pre-renal AKI and 12.77 (4.34-22.92) ng/mg in intra-renal AKI (p=<0.001). In terms of progression in patients without UTI, median uNGAL levels were 71.9 (34.7-759) ng/ml in transient AKI (reversible within 48 hours) and 455.7 (83.8-1224.2) ng/ml in persistent AKI (p=0.056), with median uNGAL/uCreatinine values of 1.39 (0.40-9.24) ng/mg and 6.52 (1.38-16.09) ng/mg in transient and persistent cases respectively (p=0.029).

**Conclusions**

Results from our local experience confirmed that uNGAL testing is useful in the diagnostic assessment of new-onset AKI. Clinical context, such as the presence of infection or UTI should be considered in interpretation of uNGAL levels, which can be improved in accuracy by correction for urinary concentration/dilution. uNGAL is a useful new test for the diagnostic and prognostic assessment of AKI in hospitalized patients.

## A24. GP perspectives on enhancing integrated care at the GP-hospital interface: a pilot delphi consensus study

### Eoghan Carey^1^, Geoff McCombe^1^, John Broughan^1^, Ronan Fawsitt^2^, Aine Carroll^1^, Walter Cullen^1^

#### ^1^UCD School of Medicine, Dublin, Ireland; ^2^Ireland East Hospital Group, Dublin, Ireland

**Background**

Ireland’s healthcare system is currently focused on delivering an integrated care system where emphasis is placed on universal healthcare which is primary care focused and patient-centred [1]. The GP-Hospital interface has been identified as a key problem area and a need to account for the various professional perspectives when guiding reform is required [2].

The aim of this study is to identify structures, processes and outcomes from GPs which may be important to enhance integrated care at the GP-Hospital interface using a Delphi consensus method.

**Methods**

A pilot e-Delphi consensus study was conducted over two rounds. In Round 1, 15 participants were asked to score 32 statements, by how much they agree with their importance in enhancing integrated care at the GP-Hospital interface. Participants were also allowed to suggest their own statements. In Round 2, the 13 participants who completed Round 1 were shown the distribution of scores from Round 1 and were asked to rescore if they wished. Eleven participants completed Round 2.

**Results**

Based on the Round 1 ranking, 15 of the 32 statements met the 70% threshold for consensus. Five additional statements suggested by participants in Round 1 were added, and two statements reached the consensus threshold. The largest consensus was observed in areas such as rapid access diagnostics, direct access to specific hospital departments and improved communication between GPs and Hospitals.

**Conclusions**

These study findings highlight important elements for enhancing integrated care at the GP-Hospital interface and can inform integrated healthcare policy in Ireland and elsewhere.

**Acknowledgement:** The author would like to acknowledge funding from the Health Research Board.

**References:**

1. Burke S, Barry S, Siersbaek R, Johnston B, Ní Fhallúin M, Thomas S. Sláintecare – A ten-year plan to achieve universal healthcare in Ireland. Health Policy. 2018;122(12):1278-82.

2. Kennedy J, Culliton, M., Leahy, D. & et al. Risk Management and Quality Improvement Strategies for Patients and Healthcare Professionals in Primary and Secondary Care.: Medisec Ireland; 2016.

## A25. Frailty in haematopoietic stem cell transplant: developing tools to identify vulnerability and improve survivorship

### Kate Banks^1^, Neasa Fitzpatrick^2^, Chris Armstrong^3^, Amanda Lavan^2^, Nina Orfali^3^

#### ^1^ School of Medicine, University College Dublin, Ireland; ^2^ Mercer’s Institute for Successful Aging (MISA), St. James’ Hospital, Dublin, Ireland; ^3^ Haematology Department, St. James’ Hospital, Dublin, Ireland

**Background:**

For many patients with haematological malignancies, an Allogeneic Haemopoietic Stem Cell Transplant (HSCT) represents the only curative option available and improvements in supportive care and the advent of reduced-intensity conditioning regimes has extended the potential benefits of HSCT to older adult patients. However, traditional measures of ‘fitness’ such as chronological age and presence of co-morbidities have been shown to be poor indicators of outcome in this population [1], thus additional metrics are needed.

Frailty in aging is the physical, psychological, and cognitive manifestation of diminishing biological reserve. It leads to increased vulnerability to both internal and external stressors [2]. In older HSCT patients, markers of vulnerability for developing frailty have been shown to be predictive of treatment related and overall mortality [3].

**Methods:**

A fitness assessment encompassing eight domains of frailty was developed, incorporating the Fried Frailty phenotype, physical weakness, functional status, falls, cognition and a subjective assessment of mood.

Over the study period, 36 patients over the age of 50 were referred for consideration for transplant and were included in the study. Patients ranged in age from 51 to 71 years old. With 22% of patients being over 65 years of age.

**Results:**

When compared to data from The Irish Longitudinal Study on Aging, the patient cohort had higher levels of risk factors associated with the development of frailty including polypharmacy, co-morbidities and falls. This indicates that they are more vulnerable to developing frailty, particularly in the face of an external stressor such as a transplant.

Objective physical tests were used to indicate a patient’s level of fitness or potential frailty. 5 patients (14%) had a Timed Up and Go score which indicated that they are more likely to be physically frail than age and gender matched peers. 7 (20%) had a Normal Walking Speed less than expected for their height and gender. 2 had a Grip Strength less than expected for their BMI and gender. No correlation was found between these metrics and traditional indicators of fitness. Interestingly, performance on physical tests did correlate with haemoglobin levels on the day of the assessment in males.

**Conclusions:**

As the population ages, the number of older adults who may benefit from HSCT will increase. Older HSCT patients have significant risk factors for the development of frailty and are more vulnerable than community-dwelling individuals. Identifying frailty in older patients and incorporating strategies to boost resilience (eg. through targeted physiotherapy regimes) prior to transplant will hopefully improve survivorship.

**References:**

1.  Artz A. Biologic vs physiologic age in the transplant candidate. Hematology. 2016;2016(1):99-105.

2.  Fried L, Cohen A, Xue Q, Walston J, Bandeen-Roche K, Varadhan R. The physical frailty syndrome as a transition from homeostatic symphony to cacophony. Nature Aging. 2021;1(1):36-46.

3.  Muffly L, Kocherginsky M, Stock W, Chu Q, Bishop M, Godley L et al. Geriatric assessment to predict survival in older allogeneic hematopoietic cell transplantation recipients. Haematologica. 2014;99(8):1373-1379.

## A26. The assessment of the severity of illness to the emergency department for psychiatric assessment

### Maia Springael¹, Dr. Anne Doherty¹ ²

#### ¹ UCD School of Medicine, University College Dublin, Belfield, Dublin 4, Ireland; ² Mater Misericordiae University Hospital, Eccles St, Dublin 7, Ireland

**Background**

COVID-19 has had a profound effect on our mental health services. In a short period of time, mental health services have had to re-configure to reduce the spread of SARS-CoV-2. This has resulted in the closure of day services, reduced in-person psychiatric support and social isolation, leaving some of society’s most vulnerable in crisis.

The purpose of this study is to identify any differences in the number and severity of emergency presentations to the Emergency Department (ED).

**Methods**

The study is a retrospective review of the log of patients referred to the liaison psychiatry team at an Inner-City Dublin hospital from the ED or inpatients wards where self-harm was the reason for admission. Three time frames were chosen between January and June 2020: a baseline group (T1), lockdown (T2) and re-opening of society (T3). Severity of presentation was measured using the Threshold Assessment Grid (TAG) (n=306)[1]. Data were analysed using the application SPSS.

**Results**

There was a significant increase in self-harm presentations in T2 and T3 (T2 - 55.1% n=27 & T3 - 38.1% n=16) with the highest incidence during the first lockdown (T2), and this was statistically significant (p=0.029). Psychiatric admissions rose during the pandemic, highest in T3 with an admission rate of 26.8% (n=11) compared to baseline (19.9%, n=39 T1, p value 0.733). Substance misuse levels were high among this population, the baseline group level of substance misuse was 57.7% (n=113) and this rose to 71.4% (n=35) and 80% (n=32) in T2 and T3 respectively (p=0.008). The study found that the homeless represent 37% (n=107) of the population seen in the ED by psychiatry (0.92% of local population). This number rose during periods of lockdown and during the reopening of society to 46.9% (n=23, T2) and 47.5% (n=19, T3) respectively.

**Conclusions**

The preliminary data suggests further research is warranted to fully understand and address the impact on this population however it is clear that there is a need to strengthen and expand current mental health systems to address the ongoing mental health crisis. Through this research we demonstrated the feasibility of doing a larger and more conclusive study with the current proposed methodology.

**References**

1. Slade M, Cahill S, Kelsey W, et al. Threshold 3: the feasibility of the Threshold Assessment Grid (TAG) for routine assessment of the severity of mental health problems. Soc Psychiatry Psychiatr Epidemiol. 2001;36(10):516-21.

## A27. Dynamic/static visual acuity with head-mounted engineering for assessment of G-transition effects and spaceflight associated neuro-ocular syndrome

### Ethan Waisberg^1^, Nasif Zaman^2^, Joshua Ong^3^, Sharif Amit Kamran^2^, Andrew G. Lee, ^4,5,6,7,8,9,10,11^, Alireza Tavakkoli^2^

#### ^1^University College Dublin School of Medicine, Belfield, Dublin, Ireland; ^2^Human-Machine Perception Laboratory, Department of Computer Science and Engineering, University of Nevada, Reno, Reno, Nevada, United States; ^3^University of Pittsburgh School of Medicine, Pittsburgh, Pennsylvania, United States; ^4^Department of Ophthalmology, Blanton Eye Institute, Houston Methodist Hospital, Houston, Texas, United States; ^5^Center for Space Medicine, Baylor College of Medicine, Houston, Texas, United States; ^6^The Houston Methodist Research Institute, Houston Methodist Hospital, Houston, Texas, United States; ^7^Departments of Ophthalmology, Neurology, and Neurosurgery, Weill Cornell Medicine, New York, New York, United States; ^8^Department of Ophthalmology, University of Texas Medical Branch, Galveston, Texas, United States; ^9^ University of Texas MD Anderson Cancer Center, Houston, Texas, United States; ^10^ Texas A&M College of Medicine, Texas, United States; ^11^Department of Ophthalmology, The University of Iowa Hospitals and Clinics, Iowa City, Iowa, United States

**Background**

Since the early Shuttle missions, astronauts have reported visual acuity (VA) changes that have led to anecdotes of diminished focus and reading checklists [1]. Further investigation has led to the discovery of Spaceflight Associated Neuro-Ocular Syndrome (SANS), a distinct set of neuro-ophthalmic findings following long-duration spaceflight (LDSF) including globe flattening and hyperopic shift. Astronauts have also demonstrated reduced dynamic VA in post-flight assessments from vestibulo-ocular adaptations during G-transitions [2]. Future planetary missions will likely involve major G-transition events, as well as exposure to microgravity longer than current LDSF. To uphold astronaut health and mission performance, consistent extraterrestrial assessment of static and dynamic VA will provide close monitoring of various microgravity-induced VA changes. A compact virtual reality (VR)-based system is being developed to provide comprehensive assessment for monitoring SANS and other vision issues including diminished dynamic visual acuity in G-transitions. In this terrestrial pilot study, we test VR-based dynamic/static VA assessments to validate the VA component in a multi-modal VR-based visual function system to detect subtle visual changes during LDSF.

**Materials and Methods**

VA will be assessed in healthy terrestrial subjects with best correctable vision VA of 20/20. Subjects will be tested with mono VA assessments for both traditional laptop-based and VR-based assessments. In addition to VA data, VR-based head-orientation, eye-tracking data, and cyclopean eye direction data will be collected.

**Results**

Validation studies with VR Dynamic VA are currently underway. Mean dynamic/static VA with traditional assessment, mean dynamic/static VA with VR-based assessment, mean head-orientation and mean cyclopean eye direction will be reported and assessed statistically.

**Conclusion**

This pilot study with VR-based visual assessment plans to showcase the reliability of dynamic and static VA assessments in a single, compact VR assessment system being developed for spaceflight. Future studies will be conducted with other visual function assessments to map a multi-modal assessment of visual function during spaceflight. These assessments should also be conducted with terrestrial analogs for SANS such as strict head-down tilt bed rest. Training with VR-based Dynamic Visual Acuity may serve as a countermeasure for planetary travel that can be conducted terrestrially and during spaceflight.

**References**

1. Bloomberg JJ, Reschke MF, Clément GR, Mulavara AP, Taylor LC. Evidence Report: Risk of Impaired Control of Spacecraft/Associated Systems and Decreased Mobility Due to Vestibular/Sensorimotor Alterations Associated with Space flight. 2016 Jun. [cited 10 January 2022] https://humanresearchroadmap.nasa.gov/evidence/reports/SM.pdf

2. Peters BT, Miller CA, Brady RA, Richards JT, Mulavara AP, Bloomberg JJ. Dynamic visual acuity during walking after long-duration spaceflight. Aviat Space Environ Med. 2011 Apr;82(4):463-6. doi: 10.3357/asem.2928.2011. PMID: 21485405.

## A28. Health barriers & facilitators for older adults, a systematic review

### Aoife Semar

#### University College Dublin, Dublin, Ireland

##### **Correspondence:** Aoife Semar (aoife.semar@ucdconnect.ie)

Older adults have unique and sometimes intensive healthcare needs as well as barriers to access, making extension of eHealth care for older adults a relevant consideration. Our research goal was to perform a systematic review of the literature describing barriers and facilitators of eHealth uptake and use by older adults. 9 articles published in peer reviewed journals were included in the review which revealed 3 major thematic groups of barriers and facilitators: (1) Personal Factors including attitudes and physical & functional abilities (2) Technological Factors including technology literacy and technology design, and (3) Structural-Societal-Socioeconomic Factors. Among these themes, the most important barriers included attitudes of a lack of perceived need for eHealth with preference for traditional healthcare [1, 2, 3, 4, 5], perceived problems with privacy, safety, reliability [1, 2, 3, 5, 6, 8, 9], physical and functional issues relating to sight and hearing [1, 2, 3, 4, 5, 7], lack of basic competency in technology literacy [1, 2, 3, 7], poor hardware and software design [1, 2, 3, 4, 9], socioeconomic and financial concerns [1, 2, 3, 4, 5]. In contrast, the most common and important facilitators were belief that technology can improve life and health [2, 5, 6, 9], improved communication and support [1, 5, 6, 8], convenience, efficiency, usefulness [1, 2, 3, 6, 4, 9], training sessions focussed on technology skills and literacy [2, 4, 5], access to support from younger family members [2, 9], affordable, cost Effective, financially beneficial [3, 4, 5]. Successful implementation of a national eHealth strategy requires acknowledgement and consideration of these barriers and facilitators toward older adults uptake and use of eHealth.

**References**

1. Vergouw JW, Smits-Pelser H, Kars MC, van Houwelingen T, van Os-Medendorp H, Kort H, Bleijenberg N. Needs, barriers and facilitators of older adults towards eHealth in general practice: a qualitative study. Primary health care research & development. 2020;21.

2. Wilson J, Heinsch M, Betts D, Booth D, Kay-Lambkin F. Barriers and facilitators to the use of e-health by older adults: a scoping review. BMC public health. 2021 Dec;21(1):1-2.

3. Airola E. Learning and Use of eHealth Among Older Adults Living at Home in Rural and Nonrural Settings: Systematic Review. Journal of medical Internet research. 2021 Dec 2;23(12):e23804.

4. Cajita MI, Hodgson NA, Lam KW, Yoo S, Han HR. Facilitators of and barriers to mHealth adoption in older adults with heart failure. Computers, informatics, nursing: CIN. 2018 Aug;36(8):376.

5. Parker, S.J., Jessel, S., Richardson, J.E. and Reid, M.C., 2013. Older adults are mobile too! Identifying the barriers and facilitators to older adults’ use of mHealth for pain management. BMC geriatrics, 13(1), pp.1-8.

6. Ware P, Bartlett SJ, Paré G, Symeonidis I, Tannenbaum C, Bartlett G, Poissant L, Ahmed S. Using eHealth technologies: interests, preferences, and concerns of older adults. Interactive journal of medical research. 2017 Mar 23;6(1):e4447.

7. Wang S, Bolling K, Mao W, Reichstadt J, Jeste D, Kim HC, Nebeker C. Technology to support aging in place: Older adults’ perspectives. InHealthcare 2019 Jun (Vol. 7, No. 2, p. 60). Multidisciplinary Digital Publishing Institute.

8. Akenine U, Barbera M, Beishuizen CR, Pour MF, Guillemont J, Rosenberg A, Coley N, Mangialasche F, Salo L, Savy S, Pols AJ. Attitudes of at-risk older adults about prevention of cardiovascular disease and dementia using eHealth: a qualitative study in a European context. BMJ open. 2020 Aug 1;10(8):e037050.

9. Johnson A, Shukla N, Halley M, Nava V, Budaraju J, Zhang L, Linos E. Barriers and facilitators to mobile health and active surveillance use among older adults with skin disease. Health Expectations. 2021 Oct;24(5):1582-92.

## A29. A correlative assessment of the role of stroma mechanics in pancreatic cancer progression

### Chris Glynn^1,2^, Michelle Fox^1,2^, Stephen D Thorpe^1,2^

#### ^1^UCD School of Medicine, University College Dublin, Belfield, Dublin 4, Ireland; ^2^UCD Conway Institute of Biomolecular & Biomedical Research, University College Dublin, Belfield, Dublin 4, Ireland

Pancreatic Ductal Adenocarcinoma (PDAC) is a highly aggressive malignancy with a low survival rate. Of all solid tumours, PDAC has the greatest amount of stromal content. The fibrous stroma is much stiffer than the cancer cell niche and is thought to drive tumour progression while promoting drug resistance. The aim of this work is to investigate the hypothesis that increased stiffness in stromal tissue is associated with increased collagen packing and alignment.

Human tissue samples from cancerous and non-cancerous regions of the pancreas were sliced and stained with Picrosirius Red before being imaged with a polarized light microscope. Polarized light microscopy allows us to image the collagen bundles within the sample by taking advantage of the birefringent properties of collagen. Each region of interest was imaged at an angle of 0, 60, 120 and 180 degrees before being constructed into a representative composite image[1]. Collagen organisation was quantified using the software CT-FIRE as well as Fourier transform-based alignment quantification software and custom MATLAB scripts [2]. Atomic Force Microscopy data from adjacent slices of our sample was supplied and compared with collagen organisation data.

Image composition and analysis was successfully carried out using a variety of contrasting techniques. Picrosirius staining intensity increased in tumour tissue relative to non-cancerous samples along with increased fibre packing evident from polarized light microscopy. Increased stiffness was observed to correlate with increased collagen content.

This work has highlighted the relationship between collagen organisation and tissue stiffness in Pancreatic Ductal Adenocarcinoma.

The author would like to acknowledge funding from Breakthrough Cancer Research and Dr Niall Swan from St. Vincent’s University Hospital for provision of pancreatic tissue sections.

**References:**

1. Greiner C, Grainger S, Farrow S, Davis A, Su JL, Saybolt MD, et al. Robust quantitative assessment of collagen fibers with picrosirius red stain and linearly polarized light as demonstrated on atherosclerotic plaque samples. PLOS ONE. 2021 Mar 18;16(3).

2. Liu Y, Keikhosravi A, Pehlke CA, Bredfeldt JS, Dutson M, Liu H, et al. Fibrillar Collagen Quantification With Curvelet Transform Based Computational Methods. Frontiers in Bioengineering and Biotechnology. 2020 Apr 21;8.

